# Novel use of FDA-approved drugs identified by cluster analysis of behavioral profiles

**DOI:** 10.1038/s41598-022-10133-y

**Published:** 2022-04-21

**Authors:** Sara Tucker Edmister, Thaís Del Rosario Hernández, Rahma Ibrahim, Cameron A. Brown, Sayali V. Gore, Rohit Kakodkar, Jill A. Kreiling, Robbert Creton

**Affiliations:** 1 Department of Molecular Biology, Cell Biology and Biochemistry, Brown University, Providence, Rhode Island USA; 2 Center for Computation and Visualization, Brown University, Providence, Rhode Island USA

**Keywords:** Biological techniques, Drug discovery

## Abstract

Repurposing FDA-approved drugs is an efficient and cost-effective approach in the development of therapeutics for a broad range of diseases. However, prediction of function can be challenging, especially in the brain. We screened a small-molecule library with FDA-approved drugs for effects on behavior. The studies were carried out using zebrafish larvae, imaged in a 384-well format. We found that various drugs affect activity, habituation, startle responses, excitability, and optomotor responses. The changes in behavior were organized in behavioral profiles, which were examined by hierarchical cluster analysis. One of the identified clusters includes the calcineurin inhibitors cyclosporine (CsA) and tacrolimus (FK506), which are immunosuppressants and potential therapeutics in the prevention of Alzheimer’s disease. The calcineurin inhibitors form a functional cluster with seemingly unrelated drugs, including bromocriptine, tetrabenazine, rosiglitazone, nebivolol, sorafenib, cabozantinib, tamoxifen, meclizine, and salmeterol. We propose that drugs with ‘CsA-type’ behavioral profiles are promising candidates for the prevention and treatment of Alzheimer’s disease.

## Introduction

Drug repurposing has been proposed for the treatment of various diseases, including cancer, neurodegenerative disorders, and Alzheimer’s disease^1–3^. Repurposing is cost-effective and efficient since existing drugs have already passed clinical trials to evaluate safety. New use may be found for drugs that have been approved by the U.S. Food and Drug Administration (FDA), as well as ‘failed’ drugs that passed phase I clinical trials to evaluate safety, but were not effective in phase II or III clinical trials. In this latter group, patent protection can provide financial incentives for additional clinical trials to examine a new use^4^. FDA-approved drugs with expired patents may be viable as well and new use can be evaluated in population studies, before initiating additional clinical trials. There is a rich history of successful drug repurposing, which includes classical examples such as acetylsalicylic acid (Aspirin) and sildenafil (Viagra)^5^. Acetylsalicylic acid was initially marketed by Bayer in 1899 for pain relief and was repurposed in the 1980s to prevent heart attacks. Sildenafil was studied in 1985 by Pfizer for the treatment of high blood pressure but was repurposed and marketed in 1998 for erectile dysfunction. In 2005, it was repurposed again for pulmonary hypertension^5^. Drug repurposing is a particularly powerful approach for small-molecule treatment of neural disorders. Small-molecules are more likely to pass the blood–brain barrier than protein-based biologics, which are typically excluded from the brain^6,7^. In addition, many molecular targets located in visceral systems are also present in the brain. For example, angiotensin receptors in blood vessels are prime targets for blood pressure medication. These receptors are also found in neurons, astrocytes, oligodendrocytes, and microglia in the brain, indicating that angiotensin inhibitors may be reused for the treatment of neural disorders^8^.

Alzheimer’s disease has been a particular challenging disorder for drug development, with many candidate drugs failing in clinical trials^9,10^. These setbacks may be caused in part by the selection of molecular targets that play a role in late neurodegenerative processes, rather than the early signaling pathways that cause Alzheimer’s disease. One of the early signaling proteins that is thought to play a key role in Alzheimer’s disease is the calcium-dependent serine-threonine phosphatase calcineurin. The following model has been proposed^11^: Intracellular free calcium increases in the aging brain due to oxidative stress, mitochondrial dysfunction and amyloid β oligomers that bind transmembrane proteins. A subtle, but prolonged, increase in intracellular calcium activates the phosphatase calcineurin. Calcineurin removes the phosphate group of signaling proteins, such as the nuclear factor of activated T-cells (NFAT), glycogen synthase kinase-3 (GSK-3), and BCL2-associated death protein (BAD). These signaling proteins then activate molecular and cellular processes associated with Alzheimer’s disease^11^. Based on this model, calcineurin inhibitors are considered viable therapeutics for early stage Alzheimer’s disease. This idea is supported by population studies, which show that transplant patients treated with the calcineurin inhibitors cyclosporine A (CsA) or tacrolimus (FK506) rarely develop Alzheimer’s disease in all age groups above 65^12^. In transplant medicine, CsA and FK506 are used as immunosuppressants to prevent organ rejection. Small molecules that are similar to CsA and FK506 in their effect on the brain, but do not suppress the immune system, may be ideal candidates for the treatment of Alzheimer’s disease. However, prediction of function is difficult for small molecules, especially in the brain. Small molecules often have multiple molecular targets and can affect interacting signaling pathways in complex neural networks. The analysis of behavior in animal model systems offers a solution, since subtle changes in neural function can be detected, even when the brain seems unaffected by molecular or structural criteria.

In the current study, we examine behavior in zebrafish larvae. Zebrafish are a popular model system in the biomedical sciences^13^ and are a particularly promising model for human brain disorders^14^. Zebrafish larvae can be imaged in vivo in microplates and specific behaviors can be measured by automated image analysis^15–23^. Moreover, high-throughput analyses of behavior have been used to screen small-molecule libraries, which led to the discovery of novel drugs with clinical relevance^13,21–25^. We treated larvae with FDA-approved drugs and measured a broad range of behaviors, including activity, habituation, acoustic startle responses, excitability, and optomotor responses. These behaviors in zebrafish are not directly linked to the behavior of people with Alzheimer’s disease. However, zebrafish larval behaviors are regulated by a wide variety of neural signaling pathways, including calcineurin signaling pathways^26^. Drugs that target these pathways will affect specific parameters of zebrafish behavior and may be used to target similar signaling pathways in human brain disorders. The zebrafish behaviors were summarized in behavioral profiles, which were examined for similarity by hierarchical cluster analysis. This cluster analysis revealed a number of seemingly unrelated drugs with ‘CsA-type’ behavioral profiles.

## Results

### Analysis of behavior

**Figure 1:**
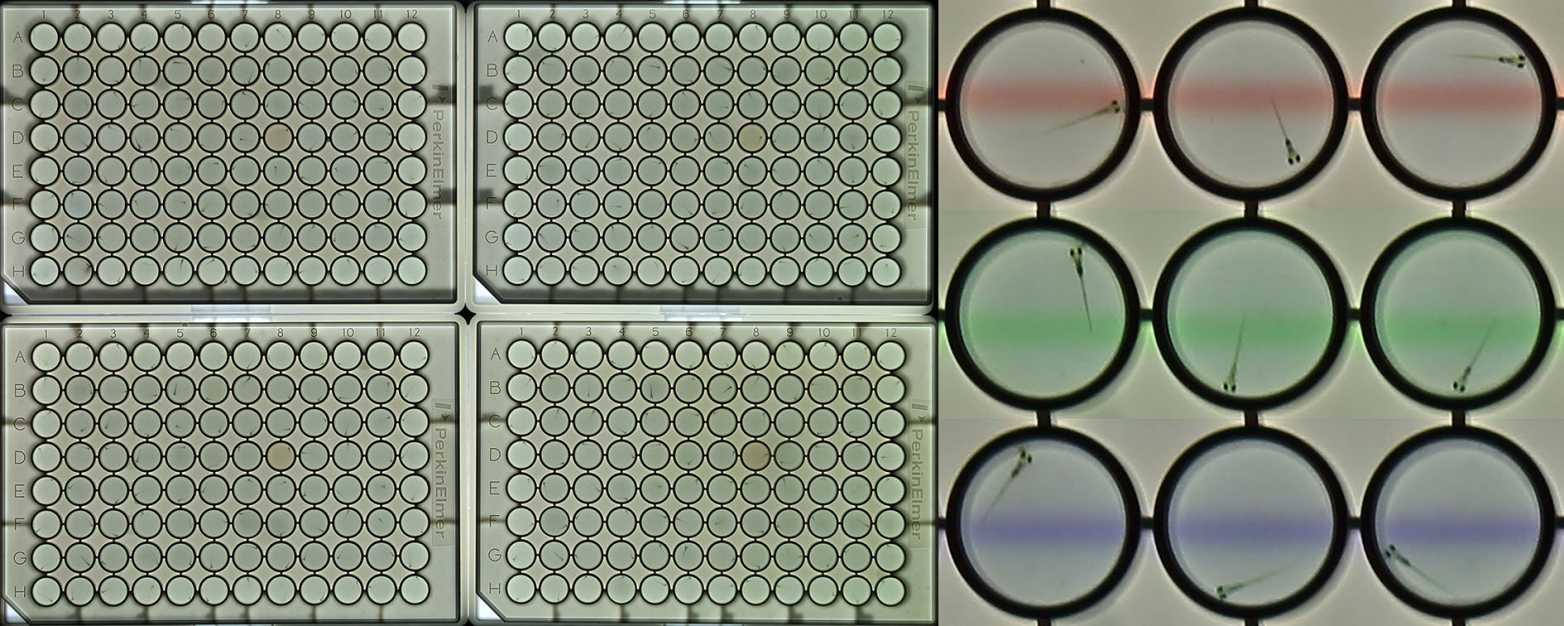
Imaging behavior in 5-day-old zebrafish larvae. The larvae are imaged in four 96-well plates for automated analysis of behavior in a 384-well format. The cropped panels on the right show three visual stimuli projected through the bottom of the plates. The red, green and blue lines move down or up in subsequent 10-min periods. Zebrafish larvae typically swim in the same direction as the lines, called an optomotor response or OMR. Larval movements and locations are measured by automated image analysis. Inner diameter of well = 7.15 mm.

**Figure 2:**
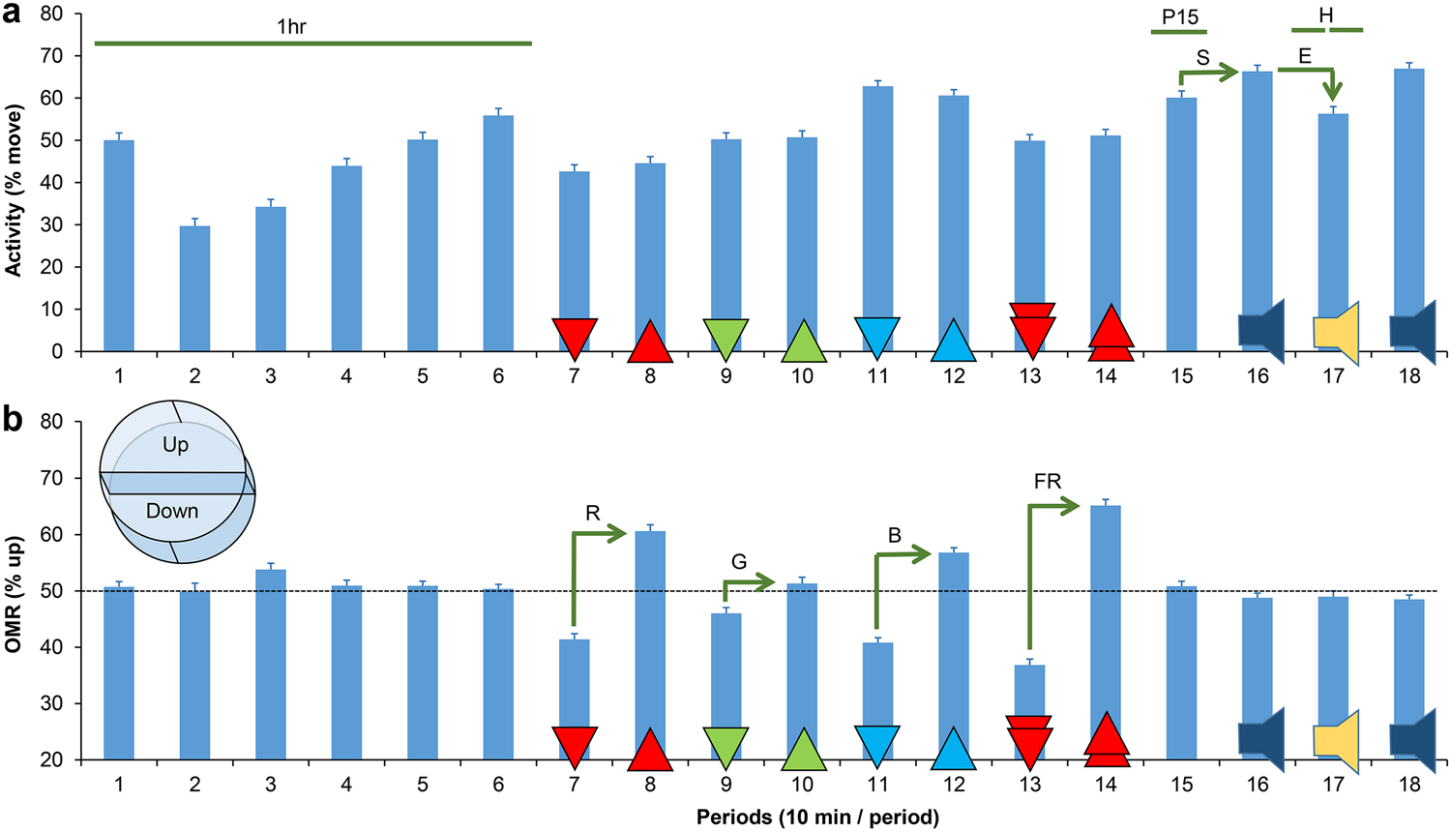
Analysis of behavior. (**a**) Activity. (**b**) Optomotor responses (OMR). Larval behaviors were examined in eighteen 10-min periods (3 h total) with and without stimuli. Period 1–6: without visual or acoustic stimuli. Period 7–8: red lines moving down and up. Period 9–10: green lines moving down and up. Period 11–12: blue lines moving down and up. Period 13–14: red lines moving down and up at a 16 × higher speed. Period 15: without visual or acoustic stimuli. Period 16: acoustic pulses at 20-s intervals. Period 17: acoustic pulses at 1-s intervals. Period 18: acoustic pulses at 20-s intervals. Values of larval activity and location were used to calculate the following 10 parameters of behavior. 1 h = average activity in period 1–6. P15 = average activity in period 15. Hab = Habituation to acoustic stimuli at 1-s intervals (first vs. second 5 min of period 17). S = Startle in response to acoustic stimuli at 20-s intervals. E = Excitability in response to acoustic stimuli at 1-s intervals. R = Optomotor response (OMR) using moving red lines. G = OMR using moving green lines. B = OMR using moving blue lines. FR = OMR using red lines, moving 16 × faster than all other lines. RGB = combined OMR using moving lines of any color or speed. The graphs show the averages and standard error or the mean for DMSO-vehicle controls (N = 384 larvae). The 3D drawing in panel b illustrates the designation of areas in a well (up = the upper half of a well in a horizontal plane).

Zebrafish larvae were imaged in four 96-well plates (Fig. 1). Larval behaviors were recorded for 3 h with various visual and acoustic stimuli. A PowerPoint file with the stimuli is included in the supplementary information (S1). Acquired images were analyzed using a custom-developed ImageJ macro, included in the supplementary information (S2). Values for larval activity and location were then calculated using two Excel templates for data processing, which are also included in the supplementary information (S3, S4). In the data processing, the 3-h recordings were divided into eighteen 10-min periods, which were used to calculate the primary outcome measures of 10 behaviors (Fig. 2).

### Effects of FDA-approved drugs

Zebrafish larvae were treated with 190 compounds using a Tocris library with FDA-approved drugs. This library was provided in three plates with 80 (plate T1), 80 (plate T2), and 30 (plate T3) stocks dissolved in DMSO at 10 mM concentrations. We carried out 12 imaging sessions for each of the three Tocris plates (36 imaging sessions in total). In each imaging session, we imaged four 96-well plates, containing four wells with duplicate drug treatments (one per plate). In our primary screen, a total of 10,944 larvae were examined, including 960 untreated larvae, 864 DMSO-vehicle control larvae, and 9,120 larvae treated with small-molecule drugs (190 compounds × 48 larvae per compound). Larvae that did not move throughout the experiment were automatically excluded in the data processing, leaving 952 untreated larvae and 844 DMSO control larvae for the analysis of behavior.

In the primary screen, we used a single concentration (10 µM) for all compounds in the library. For many pharmaceuticals, this 10 µM concentration is high in comparison to plasma concentrations of people who use these pharmaceuticals. The rationale for starting the primary screen with a single 10 µM concentration is threefold; (1) A single concentration limits the number of treatment groups, which is critical in high-throughput screening. (2) Higher doses may be needed in zebrafish than in people, due to differences between species and the duration of the treatments. (3) Relatively high concentrations can facilitate the classification of drugs, even if lower concentrations are used in medicine. There are drawbacks to this approach as well. For example, it is possible that the screen does not identify a number of drugs with interesting effects on behavior by screening at a concentration that is too high or too low.

**Figure 3:**
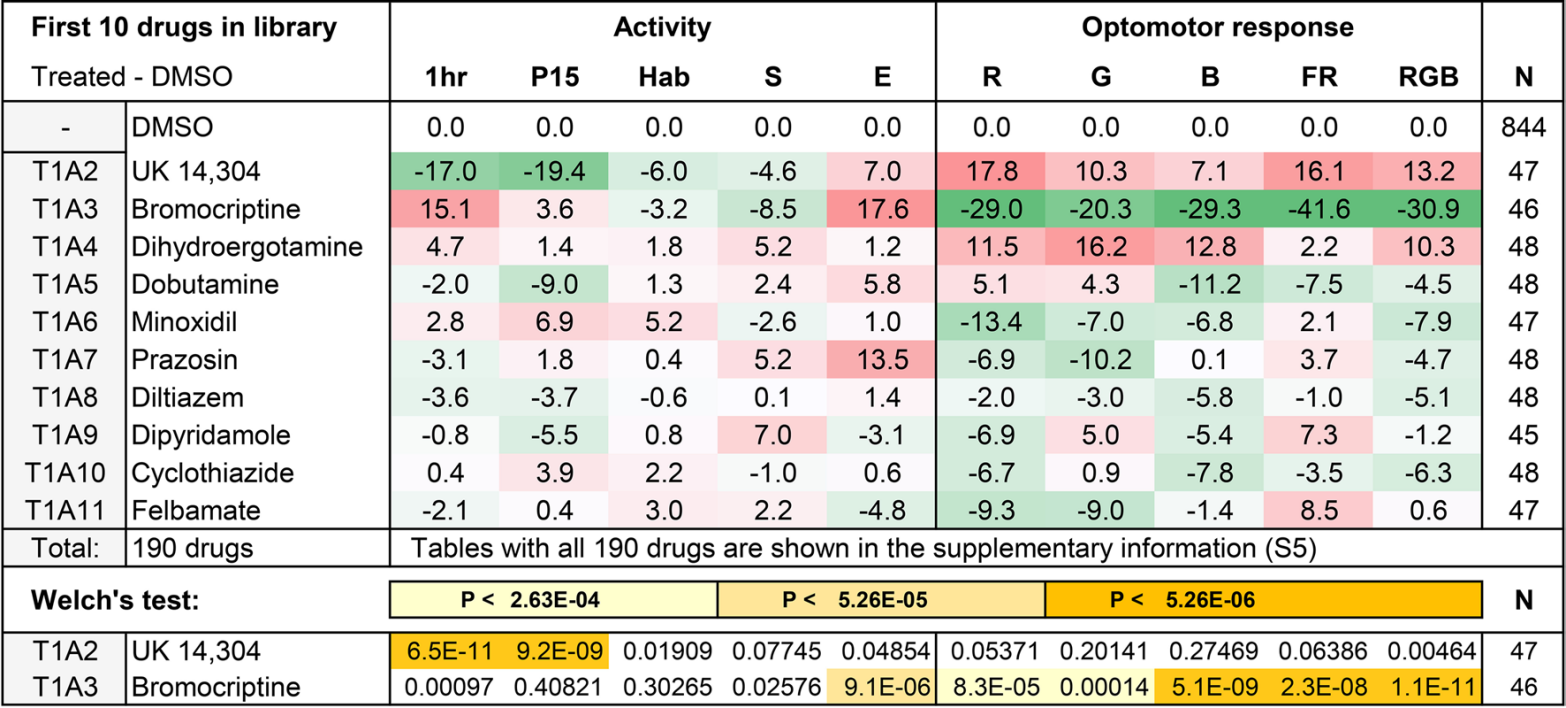
Color-coded behavioral profiles. Differences as compared to the DMSO controls were color-coded by conditional formatting to provide an overview of the changes in behavior. The colors are shown in a gradient from green (25% decrease) to red (25% increase). White indicates no change in behavior as compared to the DMSO controls. Note the decrease in activity and the increase in optomotor responses (OMR) in T1A2, which are larvae treated with UK 14,304, an adrenergic alpha-2 receptor agonist. An increase in early activity (1 h), increase in excitability (E), and decrease in OMR was detected in T1A3, which are larvae treated with bromocriptine. Treatments are identified by the Tocris plate number and well number. Differences, as compared to the DMSO control, were tested for significance using Welch’s test with a Bonferroni correction for multiple comparisons, indicating p < 2.6 × 10^–4^ (0.05/190), p < 5.3 × 10^–5^ (0.01/190), and p < 5.3 × 10^–6^ (0.001/190). The figure shows the first two rows of the statistical analysis. The remaining 8 rows did not identify any significant effects. N = number of larvae. Larvae were automatically excluded from the analysis when moving less than 1% of the time during the 3-h imaging experiment. In these cases, N < 48. The effects on behavior and statistics for all 190 compounds are provided in the supplementary information (S5).

At 10 µM concentrations, none of the treatments induced a complete loss of movement in all larvae. Thus, we were able to collect behavioral data for all 190 compounds. To create an overview of the changes in behavior, we generated ‘behavioral profiles’ by calculating differences in behavior in comparison to the DMSO-vehicle controls (Fig. 3). These differences were color-coded by conditional formatting (green = 25% point decrease, red = 25% point increase). This analysis automatically highlights large changes in behavior. For example, we found a large decrease in activity and large increase in optomotor responses in group T1A2 (UK 14,304, an adrenergic alpha-2 receptor agonist). In contrast, we found a large increase in activity and large decrease in optomotor responses in group T1A3 (bromocriptine, a non-selective dopamine agonist). Changes in behavior, as compared to the DMSO controls, were examined for statistical significance (Welch’s test with a correction for multiple comparisons). In the examples above, UK 14,304 induced a significant decrease in activity during the first hour of imaging (p = 7 × 10^–11^) and during period 15 (p = 9 × 10^–9^). Bromocriptine induced a significant increase in larval excitability (p = 9 × 10^–6^) and a significant decrease in optomotor responses (p = 8 × 10^–5^ for OMR red, p = 1 × 10^–4^ for OMR green, 5 × 10^–9^ for OMR blue, 2 × 10^–8^ for OMR fast red, and p = 1 × 10^–11^ for OMR in all colors combined). In total, 51 of the 190 FDA-approved drugs (27%) induced significant changes in behavior with p-values < 2.6 × 10^–4^ (0.05/190). The datasets and statistics for all 190 compounds are included in the supplementary information (S5).

### Hierarchical cluster analysis

**Figure 4:**
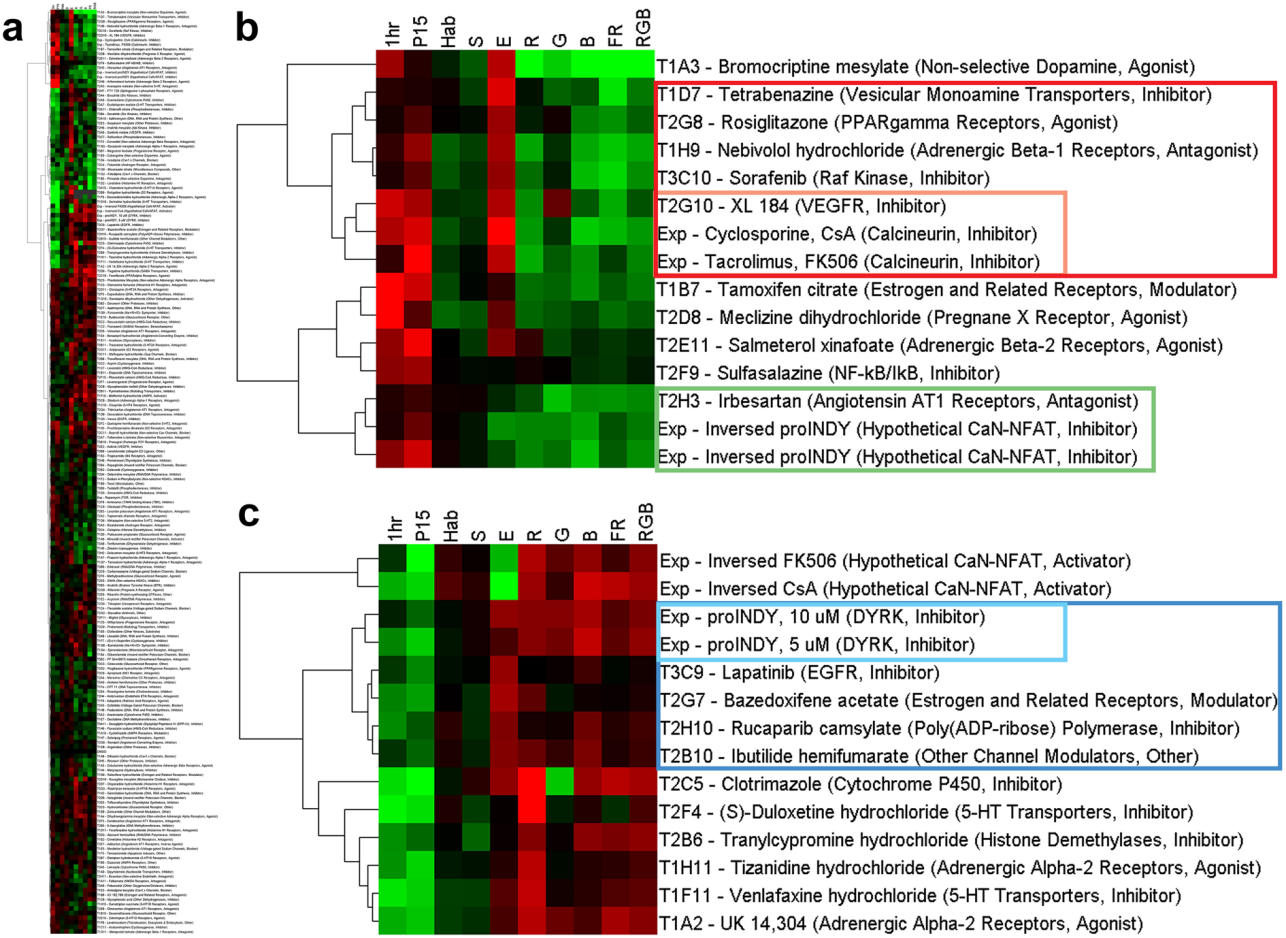
Hierarchical cluster analysis of behavioral profiles. (**a**) Overview of all screened compounds. A high-resolution image is included in the supplementary information (S6). (**b**) Large CsA-type cluster (correlation = 0.86), containing 11 drugs that group together with the calcineurin inhibitors cyclosporine (CsA) and tacrolimus (FK506). This cluster also includes the additive inverse of proINDY, which was used as a hypothetical drug to search for inhibitors of the calcineurin-NFAT signaling pathway. Red box = CsA cluster with CsA, FK506 and five other FDA-approved drugs (correlation = 0.96). Orange box = CsA cluster with CsA, FK506 and XL184 (correlation = 0.97). Green box = cluster with the additive inverse of 5 and 10 µM proINDY and Irbesartan (correlation = 0.98). (**c**) Large INDY-type cluster (correlation = 0.86), containing 10 FDA-approved drugs that cluster with the DYRK inhibitor proINDY and the additive inverse of CsA and FK506. Blue box = INDY cluster with 5 and 10 µM proINDY and four FDA-approved drugs (correlation = 0.97). Cyan box = INDY cluster with 5 and 10 µM proINDY (correlation = 0.99). Quantification of behavior: green = 25% point decrease, red = 25% point increase, black = no change, as compared to the DMSO vehicle control. Exp = prior experiments with modulators of the calcineurin (CaN) signaling pathway^26^.

Behavioral profiles are well suited for hierarchical cluster analysis. This analysis uses the magnitude of effect to reveal clusters of compounds with similar effects on behavior (Fig. 4 and supplementary information S6). Behavioral profiles of compounds in the Tocris library were combined with data sets on calcineurin signaling obtained in a prior study^26^. We found that the calcineurin inhibitors cyclosporine (CsA) and tacrolimus (FK506) form a large functional cluster with 11 seemingly unrelated drugs: (1) bromocriptine, a non-selective dopamine agonist, (2) tetrabenazine, a vesicular monoamine transporter inhibitor, (3) rosiglitazone, a PPAR-gamma receptor agonist, (4) nebivolol, an adrenergic beta-1 receptor antagonist or ‘beta-blocker’, (5) sorafenib, a Raf kinase inhibitor, (6) cabozantinib (XL184), an inhibitor of VEGFR, the vascular endothelial growth factor receptor, (7) tamoxifen, a modulator of estrogen and related receptors, (8) meclizine, a Pregnane X receptor agonist and antihistamine, (9) salmeterol xinafoate, an adrenergic beta-2 receptor agonist, (10) sulfasalazine, a NF-kB/IkB inhibitor, and (11) irbesartan, an angiotensin AT1 receptor antagonist. We refer to this class of compounds as ‘CsA-type’ drugs, which are characterized by their effect on brain function, instead of a compound’s molecular structure or previously identified target. The behavioral profiles in this large CsA-type cluster have a correlation value of 0.86.

Within the large CsA cluster, various sub-clusters can be identified. The cluster analysis revealed a close correlation (0.96) between CsA, FK506 and the following five drugs: tetrabenazine, rosiglitazone, nebivolol, sorafenib, and cabozantinib. A particularly tight correlation (0.97) was observed between CsA, FK506 and cabozantinib (XL184).

We also searched for CsA-type drugs that specifically inhibit calcineurin-NFAT signaling, instead of calcineurin signaling in general. For this search, we made use of previously obtained behavioral profiles induced by proINDY^26^. ProINDY activates NFAT via the inhibition of an inhibitor (DYRK1A) and induces behaviors that are nearly opposite to the CsA-induced behaviors. We created a hypothetical NFAT inhibitor by taking the additive inverse of all proINDY-induced behaviors (+ and − are switched). We found that inversed proINDY appears within the CsA-type cluster (Fig. 4), showing that the additive-inverse approach can be used to identify drugs with CsA-type effects on the brain. Inversed proINDY clusters particularly closely with irbesartan (correlation = 0.98).

The cluster analysis also revealed a large group of 10 drugs (correlation = 0.86) that cluster with proINDY (Fig. 4). ProINDY is an Inhibitor of DYRK, which activates calcineurin-NFAT signaling^27^. The drugs with ‘INDY-type’ effects on neural function are: (1) lapatinib, an EGFR inhibitor, (2) bazedoxifene, a modulator of estrogen and related receptors, (3) rucaparib, a poly ADP-ribose polymerase inhibitor, (4) ibutilide, other channel modulator, (5) clotrimazole, a cytochrome P450 inhibitor, (6) duloxetine, a 5-HT transporter inhibitor, (7) tranylcypromine, a histone demethylase inhibitor, (8) tizanidine, an adrenergic alpha-2 receptor agonist, (9) venlafaxine, a 5-HT transporter inhibitor, and (10) UK 14,304, an adrenergic alpha-2 receptor agonist. This INDY cluster also includes the additive inverse of CsA and FK506, again suggesting that the additive inverse approach can be used in the discovery of drugs with opposite effects. INDY-type drugs may have beneficial effects in people with Down syndrome, who have suppressed calcineurin-NFAT signaling pathways due to two genes located on chromosome 21^27–30^. However, little is known about the effects of INDY-type drugs on brain development and more detailed basic studies are needed before initiating clinical trials.

### CsA-type drugs

**Figure 5:**
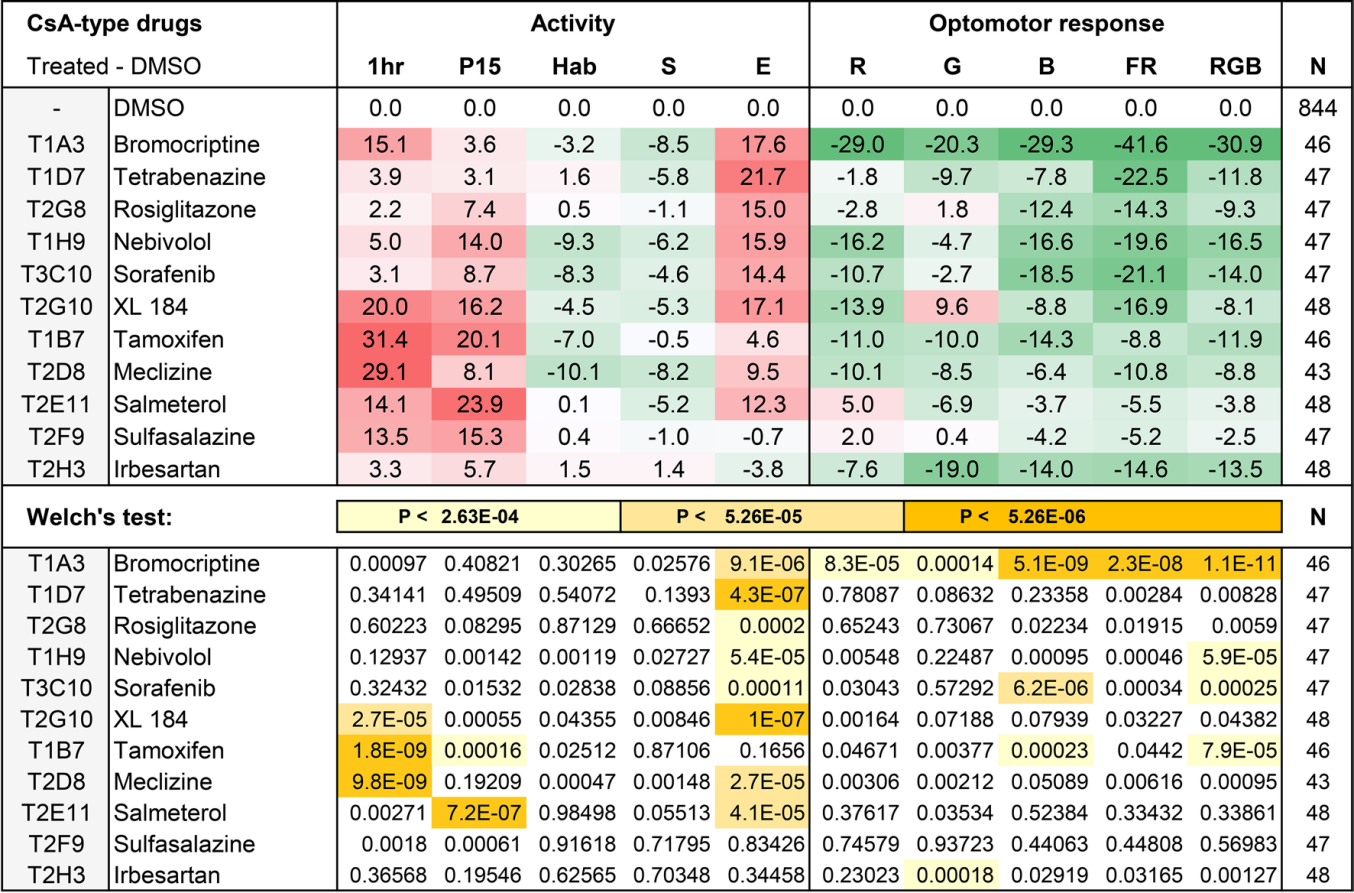
Effects of CsA-type compounds in the Tocris screen. The magnitude of the effects was color-coded in a gradient from green (25% point decrease) to red (25% point increase). White = no effect. Differences, as compared to the DMSO control, were tested for significance using Welch’s test with a Bonferroni correction for multiple comparisons, indicating p < 2.6 × 10^–4^ (0.05/190), p < 5.3 × 10^–5^ (0.01/190), and p < 5.3 × 10^–6^ (0.001/190). N = number of larvae.

The effects of CsA-type drugs in the Tocris screen and the statistical analyses of these effects are shown in Fig. 5. As compared to the DMSO controls, CsA-type drugs typically induce an increase in activity during the first hour (1 h) and period 15 (P15), an increase in excitability (E), and a decrease in optomotor responses. All CsA-type compounds induce statistically significant effects in at least one parameter of behavior, except for sulfasalazine. Sulfasalazine shows a trend toward hyperactivity, but this trend is not significant after correction for multiple comparisons. Similarly, other compounds show trends that do not reach our stringent criterion for statistical significance; p < 2.6 × 10^–4^ (0.05/190). For example, bromocriptine-treated larvae are 15% points more active than the DMSO controls during the first hour of imaging (1 h) with a p-value of 0.00097. This behavior is considered a trend, as it does not reach statistical significance. To evaluate such changes in more detail, we carried out validation experiments for all CsA-type compounds.

### Validation experiments

For the validation experiments, we ordered 11 CsA-type drugs from different vendors and created new stock solutions in DMSO. We loaded 96-well plates with 8 rows of 12 experimental groups (DMSO vehicle control and 11 CsA-type compounds), imaged 4 plates per day, and repeated the experiment 5 times (160 larvae per group, 1,920 larvae in total). After automated removal of immobile larvae in the data processing, we had the following number of larvae in the analysis: 150 (DMSO), 155 (bromocriptine), 153 (tetrabenazine), 154 (rosiglitazone), 156 (nebivolol), 147 (sorafenib), 153 (cabozantinib/XL184), 145 (tamoxifen), 154 (meclizine), 154 (salmeterol), 153 (sulfasalazine), and 152 (irbesartan). The statistical test (Welch’s test) had more power in the validation experiments than in the primary screen, in part because we used more larvae in the validation experiments, and in part, because we only compared 11 groups to the DMSO controls. After the Bonferroni correction, statistical significance is reached at p < 4.5 × 10^–3^ (0.05 / 11), p < 9.1 × 10^–4^ (0.01 / 11), and p < 9.1 × 10^–5^ (0.001 / 11).

Bromocriptine induced the following significant effects as compared to the DMSO controls; a 25% increase in 1 h activity (p = 5 × 10^–16^), a 10% increase in P15 activity (p = 3 × 10^–3^), a 7% decrease in startle responses (p = 3 × 10^–3^), a 17% increase in excitability (p = 2 × 10^–8^), a 19% decrease in OMR green (p = 1 × 10^–5^), a 20% decrease in OMR blue (p = 3 × 10^–6^), a 41% decrease in OMR fast red (p = 4 × 10^–16^), and a 21% decrease in OMR RGB (p = 4 × 10^–11^). Thus, bromocriptine-treated larvae only showed a trend of hyperactivity in the primary screen (Fig. 5), but were significantly hyperactive in the validation experiments. Similarly, bromocriptine-treated larvae only showed a trend towards reduced startle responses in the primary screen (Fig. 5), but displayed significantly reduced startle responses in the validation experiments. In the primary screen, excitability was significantly increased and optomotor responses were significantly decreased (Fig. 5). These effects were again significant in the validation experiments.

Other CsA-type drugs induced similar changes in behavior, confirming previously observed results at a higher confidence limit. Tetrabenazine induced a 20% increase in excitability (p = 2 × 10^–9^). Rosiglitazone induced an 11% increase in 1 h activity (p = 1 × 10^–3^), a 15% increase in P15 activity (p = 1 × 10^–5^), a 7% decrease in OMR blue (p = 1 × 10^–3^), and a 7% decrease in the OMR of all colors combined (p = 5 × 10^–4^). Nebivolol induced 10% increase in 1 h activity (p = 1 × 10^–4^), a 12% decrease in OMR red (p = 2 × 10^–3^), an 18% decrease in OMR fast red (p = 7 × 10^–5^), and 12% decrease in the OMR of all colors combined (p = 3 × 10^–6^). Sorafenib induced a 17% increase in excitability (p = 2 × 10^–7^), an 18% decrease in OMR blue (p = 6 × 10^–5^), a 22% decrease in OMR fast red (p = 4 × 10^–5^), and a 12% decrease in the OMR of all colors combined (p = 3 × 10^–4^). Cabozantinib (XL184) induced a 19% increase in 1 h activity (p = 9 × 10^–9^), and a 14% increase in excitability (p = 4 × 10^–7^). Tamoxifen induced a 13% increase in 1 h activity (p = 5 × 10^–5^), an 18% decrease in OMR blue (p = 2 × 10^–5^), a 22% decrease in OMR fast red (p = 1 × 10^–6^), and a 14% decrease in the OMR of all colors combined (5 × 10^–7^). Meclizine induced a 29% increase in 1 h activity (3 × 10^–19^), a 13% increase in P15 activity 3 × 10^–4^), an 8% decrease in habituation (p = 3 × 10^–3^), an 8% decrease in startle responses (p = 5 × 10^–4^), an 11% decrease in OMR red (4.5 × 10^–3^), a 19% decrease in OMR blue (6 × 10^–7^), a 14% decrease in OMR fast red (p = 2 × 10^–3^), and a 14% decrease in the OMR of all colors combined (p = 9 × 10^–8^). Salmeterol induced a 19% increase in 1 h activity (p = 1 × 10^–8^), and a 21% increase in P15 activity (p = 2 × 10^–9^). Other behaviors were not significant and occasionally showed opposite effects in the primary screen and validation experiments. For example, tetrabenazine and tamoxifen showed a 9% and 11% decrease in period 15 activity (p = 4 × 10^–3^, 3 × 10^–3^). Sulfasalazine and irbesartan did not induce any significant effects in the validation experiments and the observed trends were conflicting in the primary screen and the validation experiments. Sulfasalazine-treated larvae displayed a trend of hyperactivity in the primary screen (Fig. 5). In the validation experiments, this trend was still observed but did not reach statistical significance. Irbesartan-treated larvae displayed a significant reduction of the optomotor response in the primary screen (OMR green). However, none of the OMRs decreased significantly in the validation experiments. In addition, irbesartan-treated larvae displayed an opposite trend in the validation experiments with a 12% increase in OMR red (not significant at p = 0.01). In summary, 9 out of 11 CsA-type drugs (82%) induced changes in behavior that were repeatable and significant in the validation experiments.

### Concentration series

Three CsA-type drugs were examined in more detail at 5, 10, and 20 µM concentrations. Larvae were also treated with 1 µl/ml DMSO as a vehicle control (the same concentration of DMSO was present in all treatment groups). We focused on bromocriptine, nebivolol, and cabozantinib (XL184). Bromocriptine was selected because of its striking effect on the optomotor response (Fig. 5). Nebivolol was selected because its broad use as a beta-blocker and potential use in the treatment of Alzheimer’s disease^31^. Cabozantinib was selected because of its similarity to cyclosporine in the cluster analysis (Fig. 4). In each case, treated groups were compared to the DMSO vehicle controls and differences between these two groups were tested for significance using Welch’s test. After the Bonferroni correction, statistical significance is reached at p < 0.017 (0.05/3), p < 3.3 × 10^–3^ (0.01/3), or p < 3.3 × 10^–4^ (0.001/3).

Bromocriptine was examined at 0, 5, 10, and 20 µM concentrations (n = 94, 96, 96, 96 larvae). These concentrations induced various changes in behavior, as compared to the DMSO controls. Treatment with 5, 10, and 20 µM bromocriptine induced a 13%, 27%, and 52% increase in 1 h activity (p = 6 × 10^–7^, p = 1 × 10^–16^, and p = 4 × 10^–45^). The 5 and 10 µM bromocriptine treatments did not induce significant changes in period 15 activity, habituation, or startle responses. In contrast, 20 uM bromocriptine induced a significant 22% increase in period 15 activity (p = 3 × 10^–8^), 9% decrease in habituation (p = 1 × 10^–3^), and 13% decrease in the startle response (p = 1 × 10^–6^). Treatment with 5, 10, and 20 µM bromocriptine induced a 17%, 32%, and 31% increase in excitability (p = 9 × 10^–6^, 8 × 10^–17^, and 1 × 10^–17^). Treatment with 10 and 20 µM bromocriptine induced an atypical 18% and 19% increase in OMR red (p = 2 × 10^–3^, 1 × 10^–3^), while treatment with 20 µM bromocriptine induced a 17% decrease in OMR green (p = 5 × 10^–4^). Treatment with 5, 10, and 20 µM bromocriptine induced a 26%, 32%, and 33% decrease in OMR fast red (p = 2 × 10^–4^, 2 × 10^–7^, and 1 × 10^–8^). Overall, significance was reached for 3 behaviors at 5 µM, 4 behaviors at 10 µM, and 8 behaviors at 20 µM concentrations.

Nebivolol was examined at 0, 5, 10, and 20 µM concentrations (n = 139, 144, 139, 142 larvae). These concentrations induced various changes in behavior, as compared to the DMSO controls. Treatment with 5, 10, and 20 µM nebivolol induced a 20%, 21%, and 10% increase in 1 h activity (p = 4 × 10^–18^, 2 × 10^–20^, 1 × 10^–9^). Treatment with 5 and 10 µM nebivolol induced an 8% and 10% increase in period 15 activity (p = 0.01, 2 × 10^–3^). In contrast, 20 µM nebivolol did not have a significant effect on period 15 activity. Treatment with 20 µM nebivolol induced a 9% decrease in habituation (p = 2 × 10^–4^). Treatment with 5, 10, and 20 µM nebivolol induced a 17%, 16%, and 12% increase in excitability (p = 1 × 10^–7^, 4 × 10^–7^, 5 × 10^–5^). Treatment with 10 and 20 µM nebivolol induced a 15% and 11% decrease in OMR fast red (p = 2 × 10^–3^, 0.01) and an 8% and 7% decrease in OMR all colors combined (p = 9 × 10^–3^, 5 × 10^–3^). Overall, significance was reached in 3 behaviors at 5 µM, 5 behaviors at 10 µM, and 5 behaviors at 20 µM concentrations.

Cabozantinib (XL184) was examined at 0, 5, 10, and 20 µM concentrations (n = 156, 172, 171, and 170 larvae). These concentrations induced various changes in behavior, as compared to the DMSO controls. Treatment with 5, 10, and 20 µM cabozantinib induced a 28%, 59%, and 47% increase in 1 h activity (p = 3 × 10^–18^, 4 × 10^–72^, 3 × 10^–46^) and induced a 8%, 32%, 28% increase in period 15 activity (p = 9 × 10^–3^, 2 × 10^–23^, 2 × 10^–18^). Treatment with 10 and 20 µM induced a 7% and 8% decrease in habituation (p = 8 × 10^–4^, 1 × 10^–4^) and a 6% and 5% decrease in the startle response (p = 1 × 10^–3^, 7 × 10^–3^). Treatment with 5, 10, and 20 µM cabozantinib induced an 18%, 11%, and 10% increase in excitability (p = 6 × 10^–10^, 2 × 10^–5^, 8 × 10^–5^). Treatment with 5 µM induced a 15% decrease in OMR red (p = 7 × 10^–4^). Treatment with 5 and 10 µM induced a 21% and 19% decrease in OMR blue (p = 1 × 10^–6^, 1 × 10^–7^), a 34% and 22% decrease in OMR fast red (p = 8 × 10^–11^, 2 × 10^–6^), and a 19% and 14% decrease in the OMR of all colors combined (p = 2 × 10^–8^, 3 × 10^–6^). In contrast, 20 µM cabozantinib did not induce any changes in the OMR. Overall, significance was reached in 7 behaviors at 5 µM, 8 behaviors at 10 µM, and 5 behaviors at 20 µM.

Based on the concentration series, we conclude that the general CsA-type profile can be detected using different concentrations of bromocriptine, nebivolol, and cabozantinib. However, the magnitude of effects and statistical significance both depend on the concentrations of these drugs.

## Discussion

**Figure 6:**
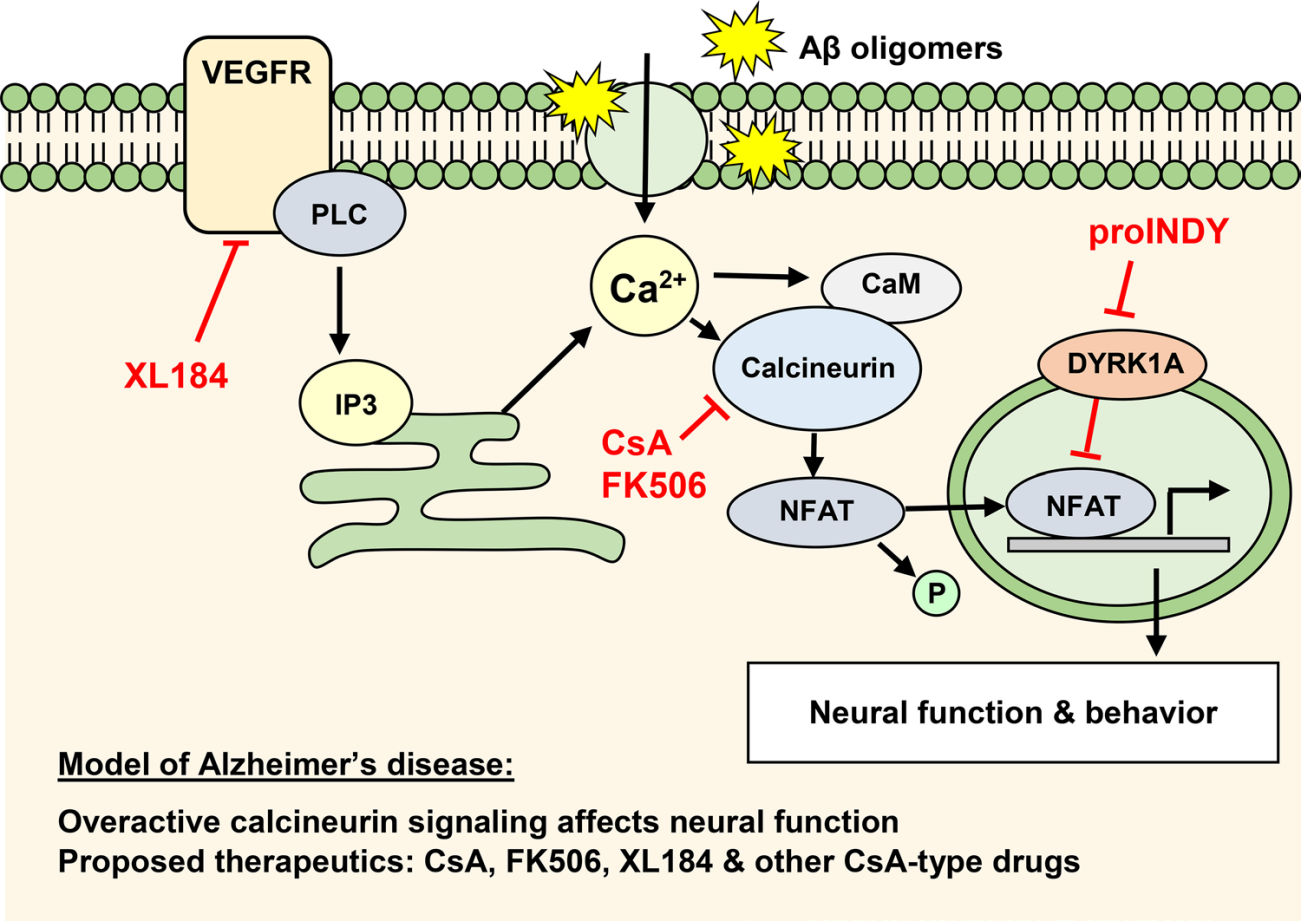
Model of interacting signaling pathways. Calcineurin-NFAT signaling can be suppressed using the calcineurin inhibitors cyclosporine (CsA) and tacrolimus (FK506). In contrast, proINDY activates calcineurin-NFAT signaling, by inhibiting an inhibitor (DYRK1A). Calcineurin inhibitors and proINDY have opposite effects on various behaviors, suggesting that calcineurin-NFAT signaling plays a key role in the regulation of neural function^26^. Various lines of evidence suggest that calcineurin signaling is activated in Alzheimer’s disease^11,12^. Oxidative stress, mitochondrial dysfunction and amyloid β (Aβ) oligomers contribute to increased intracellular free calcium (Ca^2+^), which activates calcineurin. Activated calcineurin dephosphorylates various signaling proteins, such as NFAT, BAD and GSK-3, which in turn induce various hallmarks of Alzheimer’s disease. Cabozantinib (XL184), one of the CsA-type drugs identified in the current study, is known to suppress proteins upstream of calcineurin signaling. *VEGFR* vascular endothelial growth factor receptor, *XL184* Cabozantinib, *PLC* phospholipase C, *IP3* inositol trisphosphate, *Ca*^*2+*^ intracellular free calcium.

The present study shows that a wide variety of FDA-approved drugs affect behavior. Changes in behavior were summarized in behavioral profiles, which were examined by hierarchical cluster analysis. The cluster analysis was carried out using 190 FDA-approved drugs as well as small molecules that affect the calcineurin-NFAT signaling pathway. This signaling pathway has been described in detail in the immune system during T-cell activation, but may also play a key role in the regulation of neural function and behavior (Fig. 6). The cluster analysis identified a group of 11 FDA-approved drugs with CsA-type effects on neural function. Two of these drugs, sulfasalazine and irbesartan, did not induce a CsA-type profile in the validation experiments and were removed from our list of CsA-type drugs. The following 9 CsA-type drugs were validated: bromocriptine, tetrabenazine, rosiglitazone, nebivolol, sorafenib, cabozantinib, tamoxifen, meclizine, and salmeterol.

Cabozantinib (XL184), displayed a particularly tight correlation (0.97) with the calcineurin inhibitors CsA and FK506. Cabozantinib is used for cancer treatment and inhibits various receptor tyrosine kinases, including VEGFR, MET, RET, KIT, AXL and FLT3^32^. The inhibition of VEGFR, the vascular endothelial growth factor receptor, fits well within the calcineurin signaling model (Fig. 5). VEGFR acts through phospholipase C (PLC), inositol triphosphate (IP3) and calcium (Ca^2+^) release from the endoplasmic reticulum, which activates calcineurin signaling^33^. Thus, inhibition of VEGFR likely inhibits calcineurin signaling, similar to the inhibition of calcineurin signaling with CsA or FK506. VEGFR signaling may also mediate the effects of sorafenib, another drug in the CsA-type cluster. Sorafenib is used for cancer treatment and inhibits various protein kinases, including RAF, VEGFR and PDGFR^34^. Thus, sorafenib may inhibit calcium-calcineurin signaling through VEGFR, similar to cabozantinib.

The signaling pathways affected by cabozantinib involves phospholipase C (Fig. 6). However, it would have been difficult to predict which drugs fall within the CsA-type cluster based on the signaling pathways. First, small-molecule drugs often affect multiple targets. For example, cabozantinib not only affects VEGFR, but also MET, RET, KIT, AXL and FLT3^32^. Second, many other drugs suppress VEGFR, phospholipase C and calcium signaling, but do not induce a CsA-type behavioral profile. For example, sunitinib and axitinib are two VEGFR inhibitors that were included in the small-molecule screen, but did not fall in the CsA-type cluster. Third, small-molecule drugs may affect cell signaling in vitro or in specific visceral organs, but not necessarily regulate neural function in the brain. The challenges in predicting effects highlight the complementary roles of hypothesis-driven research and unbiased high-throughput screening, which can be used to identify small molecules with surprising effects.

One of the key questions raised by the current study is the following: how can CsA-type drugs with different molecular targets have similar effects on neural function? Other than cyclosporine and tacrolimus, most of the CsA type drugs do not seem to act directly on calcineurin. It is possible that signaling pathways are affected far upstream or far downstream of calcineurin activation. In addition, multiple pathways may interact. Thus, the underlying molecular mechanisms are likely complex and may require large-scale molecular methodologies, such as single-cell RNAseq, to better understand which key processes are activated in response to the diverse group of CsA-type drugs. Further studies on CsA-type drugs are also needed to examine how this group of drugs affects neural function in other model systems, such as adult zebrafish, aging mice, Alzheimer’s model mice, and human cell cultures. Such studies could for example reveal if there are benefits to combining multiple CsA-type compounds.

Calcineurin signaling pathways have clinical relevance in Alzheimer’s disease (Fig. 6). The activation of calcineurin leads to dephosphorylation of NFAT, BAD and GSK-3, which in turn induce various hallmarks of Alzheimer’s disease^11,12^. The present study identified 9 FDA-approved drugs with CsA-type effects on neural function. We propose that these drugs are prime candidates for the prevention and treatment of Alzheimer’s disease, since these drugs are similar to CsA in their effects on neural function, but do not target the immune system. In addition, it may be possible to select specific drugs, or combinations of drugs, that are effective in the prevention and treatment of Alzheimer’s disease, without causing adverse side effects. Little is known about the potential use of CsA-type drugs such as tetrabenazine, cabozantinib, meclizine and salmeterol in Alzheimer’s disease. Other CsA-type drugs have previously been examined in Alzheimer’s models, human population studies, or clinical trials. This subset of CsA-type drugs includes bromocriptine, rosiglitazone, nebivolol, sorafenib, and tamoxifen.

Bromocriptine, a dopamine agonist used in Parkinson’s disease, reduced amyloid β peptides in iPSC-derived neurons from Alzheimer’s disease patients^35^. This promising result will be further pursued in a clinical trial in Japan^36^. Rosiglitazone, an insulin sensitizer, has been used successfully in various Alzheimer’s models and gave promising results in Phase 1 and 2 clinical trials, however, large-scale Phase 3 clinical trials did not show beneficial effects in the prevention of Alzheimer’s disease^37^. Nebivolol, a beta-blocker, reduced amyloid neuropathology in a key model for Alzheimer’s disease, the AD-model mouse^31^. Since beta-blockers are widely used for heart disease in the 50 + population, there is keen interest in determining which beta-blockers may be beneficial for the prevention of Alzheimer’s disease. Sorafenib, a Raf-kinase inhibitor used in cancer treatment, reduced neuroinflammation in AD model mice^38,39^ and has been identified as a potential therapeutic for Alzheimer’s disease by artificial intelligence^40^. The latter study also identified tamoxifen, an estrogen receptor modulator used in breast cancer treatment, as a potential Alzheimer’s disease drug^40^.

We conclude that CsA-type drugs show promising results in Alzheimer’s models and human population studies. So far, only a few compounds have been tested in clinical trials and additional clinical trials are needed to examine novel use of FDA-approved drugs in the prevention and treatment of Alzheimer’s disease.

## Materials and methods

### Approval for animal experiments

All experiments were carried out in accordance with federal regulations and guidelines for the ethical and humane use of animals and have been approved by Brown University’s Institutional Animal Care and Use Committee (IACUC). The Animal Welfare Assurance Number is D16-00183. We followed the PREPARE, 3R, and 3S guidelines when we applied for and received IACUC approval. In addition, we carried out our experiments in accordance with the ARRIVE guidelines 2.0. ARRIVE items such as sample size, randomization, blinding, and statistical methods are discussed in the sections below.

### Zebrafish

Adult wild-type zebrafish (*Danio rerio*) are maintained in the Animal Care Facility at Brown University as a genetically-diverse outbred strain in a mixed male and female population. The zebrafish are kept in a Marineland Vertical Aquatic Holding System on a 14-h light, 10-h dark cycle. The fish are fed daily with Gemma Micro 300 and frozen brine shrimp. Zebrafish embryos were collected and grown to larval stages as previously described^18,19^. Zebrafish embryos from 0–3 days post-fertilization (dpf) and zebrafish larvae from 3–5 dpf were maintained at 28.5 °C in 2L culture trays with egg water, containing 60 mg/L sea salt (Instant Ocean) and 0.25 mg/L methylene blue in deionized water (pH 7.2). Embryos and larvae were kept on a 12 h light / 12 h dark cycle and were randomly assigned to different experimental groups prior to experimental manipulation. The sex of embryos and larvae cannot be determined at such early stages because zebrafish use elusive polygenic factors for sex determination, and both males and females have juvenile ovaries between 2.5 and 4 weeks of development^41^. Zebrafish larvae were imaged at 5 dpf when the larvae display a range of locomotor behaviors and consume nutrients available in the yolk sac^42^. Larvae are approximately 4 mm long at the 5 dpf stage.

### Sample size and randomization

The experiments were designed to use the minimum number of zebrafish larvae needed for an evaluation of multiple behaviors. Sample size was determined a priori, based on analyses of behavior in prior studies^19,26^. We decided on 190 drugs, using 48 larvae for each pharmacological treatment (n = 48 larvae per drug). A smaller sample size would limit detection of subtle changes in behavior, especially for a subset of behaviors that are difficult to measure (e.g. visually-guided behaviors in response to green stimuli). In each imaging experiment, four 96-well plates were loaded with 5-dpf zebrafish larvae, transferring the larvae in a random order to avoid loading bias. For example, row B1-12 may be loaded before or after row F1-12 (for numbering of the wells, see Fig. 1). Similarly, plate 2 may be loaded before or after plate 1. We did typically finish loading a full row before moving on to the next row. Treatment solutions were then added to the wells so that each 96-well plate contained 80 wells with different drugs. Thus, there were no duplicate drug treatments within a single 96-well plate. The remaining 16 wells were used for untreated and vehicle controls. On a typical day, we transferred 384 larvae into four plates, started the treatments at 10 am, and imaged the four plates from 1–4 pm. We did not use a blinded approach for the drug treatments or subsequent analysis. Instead, we aimed to avoid bias by examining a large number of treatment groups and using automated methods for imaging, image analysis, and data processing.

### Pharmacological treatments

At 5 dpf, zebrafish larvae were incubated in treatment solutions for 3 h prior to imaging, and for 3 h during imaging, using 96-well ProxiPlates (PerkinElmer, 6006290). Zebrafish larvae were treated with 190 FDA-approved compounds using a Tocris small-molecule library (Tocris Bioscience, Cat. No. 7200). The library contained 10 mM stocks dissolved in dimethyl sulfoxide (DMSO), which we diluted 1000 × in egg water to a 10 µM final concentration. The imaging experiments also included untreated larvae in egg water and larvae treated with 1 µl/ml DMSO as a vehicle control. The effects of FDA-approved drugs were compared to previously obtained results^26^, with 10 µM cyclosporine A (CsA, Enzo Life Sciences), 1 µM tacrolimus (FK506, Enzo Life Sciences), 1 µM rapamycin (Santa Cruz Biotechnology) and 5 and 10 µM proINDY (Tocris Bioscience).

### Validation experiments and concentration series

Validation experiments were carried out using 11 CsA-type compounds purchased from a different vendor. Bromocriptine was purchased from Enzo Life Sciences (BML-D102-0100). Tetrabenazine was purchased from TCI America (T2839200MG) via Fisher Scientific. Rosiglitazone was purchased from TCI America (R0106200MG) via Fisher Scientific. Nebivolol was purchased from TCI America (N0954-20MG) via Fisher Scientific. Sorafenib was purchased from MedChemExpress (50-187-1951) via Fisher Scientific. Cabozantinib (XL184) was purchased from LC Laboratories (849217-68-1) via Fisher Scientific. Tamoxifen was purchased from Millipore Sigma (57-900-0100MG) via Fisher Scientific. Meclizine was purchased from MP Biomedicals (0215534105) via Fisher Scientific. Salmeterol Xinafoate was purchased from TCI America (A3190-250MG) via Fisher Scientific. Sulfasalazine was purchased from Fisher Scientific (AC461240050). Irbesartan was purchased from TCI America (I08591G) via Fisher Scientific. For the validation experiments, we aimed for 160 larvae per experimental group, to confirm the obtained results in the primary screen and detect more subtle effects that cannot be observed in a large-scale screen. We also examined a subset of CsA-type drugs at 0, 5, 10, and 20 µM. We focused on bromocriptine, nebivolol, and cabozantinib using the chemicals that were ordered for the validation experiments. New stocks were prepared at 5, 10, and 20 mM. This way each stock can be diluted 1000 × for the final treatment and a single 1 µl/ml DMSO control is appropriate for all treatment groups.

### Imaging system

Zebrafish larvae were imaged in an imaging system that holds four 96-well plates for automated analysis of behavior in a 384-well format (Fig. 1). The plates were imaged as described previously^19^. Briefly, the imaging system is housed in a 28.5ºC temperature-controlled cabinet where larvae in white 96-well ProxiPlates are placed onto a glass stage. Above the stage, a high-resolution camera (18-megapixel Canon EOS Rebel T6 with an EF-S 55–250 mm f/4.0–5.6 IS zoom lens) captures an image of the larvae in the four 96-well plates every 6 s. The camera is connected to a continuous power supply (Canon ACK-E10 AC Adapter) and controlled by a laptop computer using Canon’s Remote Capture software (EOS Utility, version 3), which is included with the camera. Two small speakers (OfficeTec USB Computer Speakers Compact 2.0 System) were attached speaker-side down to the glass stage. Speakers were connected by USB to the laptop computer and set to maximum volume (85 dBA). A M5 LED pico projector (Aaxa Technologies) with a 900 lumens LED light source is located below the glass stage. This projector is used for background illumination and the display of visual stimuli, using the opaque bottom of the 96-well plates as a rear projection screen.

### Behavioral assay

Visual and acoustic stimuli are controlled by an automated 3-h PowerPoint presentation that is shown to the larvae. The entire 3-h presentation has a light gray background and starts with a 1-h period without visual or acoustic stimuli, followed by 80 min of visual stimuli, a 10-min period without visual or acoustic stimuli, and 30 min with acoustic stimuli (Fig. 2). Larvae were not exposed to visual stimuli and acoustic stimuli at the same time.

The visual stimuli consisted of a series of moving lines that were red, green or blue. Prior studies have shown that zebrafish larvae will swim in the same direction as moving lines, a behavior that is called an optomotor response or OMR^19,43^. Our previously-developed assays for visually-guided behaviors indicate 5 dpf larvae consistently respond to 1 mm thick lines set 7 mm apart that move 7 mm per 8 s downwards or upwards, alternating direction in 10-min periods^19^. Additionally, the presentation included red lines that moved at a faster speed of 7 mm per 0.5 s (16 × faster). We used the following sequence of moving visual stimuli in subsequent 10-min periods: downward red lines, upward red lines, downward green lines, upward green lines, downward blue lines, upward blue lines, downward fast red lines, upward fast red lines. The brightness of the background (RGB = 210, 210, 210), red lines (RGB = 255, 0, 0), green lines (RGB = 0, 180, 0), and blue lines (RGB = 0, 0, 230) in the PowerPoint presentation are carefully matched to the camera settings (ISO200, Fluorescent, F5, 1/5 exposure) for optimal color separation in the automated image analysis.

The acoustic stimuli consisted of brief sine waves or ‘pulses’ (100 ms, 400 Hz) created in Audacity as 20-s sound tracks and inserted in the PowerPoint presentation. Larvae were first exposed for 10 min to repeated acoustic pulses with a 20-s interval, followed by 10 min of repeated acoustic pulses with a 1-s interval, and 10 min of repeated acoustic pulses with a 20-s interval. The PowerPoint presentation with the visual and acoustic stimuli is included in the Supplementary Information (S1).

### Image analysis

We developed an ImageJ macro (version 26rc062019) for automated analysis of behavior in a 384-well format^19^. This macro is available in the supplementary information (S2). The ImageJ macro can analyze four 96-well plates with multiple treatment groups. Users are prompted to enter information about the plates and the periods with different visual stimuli. The software opens the first image, splits the color channels, and selects a channel in which the visual stimuli and background have similar intensities. Subsequent images are subtracted from each other to remove the background and highlight larvae that move. The software then applies a threshold (40–255), selects the first well, measures the area and centroid of the larva and logs the measurements in a ‘Results’ file. This process is automatically repeated for all wells in an image and all subsequent images in a series. The macro calculates if a larva moved and calculates, after each movement, if a larva is located in the upper half of a well—in a horizontal plane. This upper half in a horizontal plane corresponds to the upper half in a vertical plane when looking at an acquired image on a computer screen. These measures are referred to as ‘up’, as shown in Fig. 2. The Results file of a single experiment contains approximately 10 million data points, i.e. 15 columns with information on the image, well, larval movement and larval location and 691,200 rows showing this information for each well in subsequent images (384 wells × 1800 images).

### Data processing and outcome measures

The Results files are processed in two MS Excel templates (A and B), which are included in the supplementary information (S3, S4). Template A calculates the percentage of time that a larva moves (% move) and is located in the upper half of the well (% up) in subsequent 10 min periods with various visual and acoustic stimuli (18 periods in 3 h). For the optomotor response (OMR), larval locations are compared between two 10-min periods when visual stimuli move up vs. down. Criteria for exclusion were set a priori in Excel template A. The template automatically excludes zebrafish larvae that move less than 1% of the time in a 3-h recording. In addition, larvae that move less than 5% of the time in a 10-min period are automatically excluded from OMR measurements during that period. Activity and OMR values are processed to examine the following 10 behaviors, which are the primary outcome measures of this study. (1) The average activity during the first hour of imaging without visual or acoustic stimuli. (2) The average activity in period 15 without visual or acoustic stimuli. (3) Habituation to acoustic stimuli at 1-s intervals, measured as the activity during the first 5 min minus the last 5 min of period 17. (4) Startle responses to acoustic stimuli at 20-s intervals, calculated as the activity during period 16 minus period 15. (5) Excitability in response to acoustic stimuli at 1-s intervals, calculated as the activity during period 17 minus period 16. (6) OMR using moving red lines, (7) OMR using moving green lines, (8) OMR using moving blue lines, (9) OMR using red lines, moving 16 × faster than all other lines, and (10) combined OMR using moving lines of any color or speed. A summary sheet shows the 10 behavioral parameters for each of the wells (384 rows per experiment). Template B combines the summary sheets of multiple experiments. This template calculates the average values per treatment group and calculates the differences of these groups as compared to the DMSO vehicle controls. These differences are expressed in percentage points (% points). For example, when larvae are active 10% of the time in the DMSO control and 20% of the time in a treated group, the difference is calculated as 10% points. The template tests differences between DMSO controls and treated groups for statistical significance and shows behavioral profiles in a format that is suitable for subsequent cluster analysis.

### Statistical analyses

Statistical analyses were carried out in MS Excel 2016 using Welch’s test, an unequal variances t-test. This test is well suited for the continuous data in our studies and is recommended over Student’s t-test and the non-parametric Mann–Whitney U test when the distribution is heavy tailed, sample size is unequal, and the variances may be unequal between groups^44^. A Bonferroni correction was applied for multiple comparisons. In the Tocris screen, 190 drugs were compared to the DMSO vehicle controls and differences were considered significant when p < 2.6 × 10^–4^ (0.05/190), p < 5.3 × 10^–5^ (0.01/190), or p < 5.3 × 10^–6^ (0.001/190). This conservative correction reduces the chance of a false positive, which is important in large-scale screens. Validation experiments were carried out comparing 11 treatment groups vs. a DMSO control and differences were considered significant when p < 4.5 × 10^–3^ (0.05/11), p < 9.1 × 10^–4^ (0.01/11), or p < 9.1 × 10^–5^ (0.001/11). Concentration series experiments were carried out comparing 3 concentrations vs. a DMSO control and differences were considered significant when p < 0.017 (0.05/3), p < 3.3 × 10^–3^ (0.01/3), or p < 3.3 × 10^–4^ (0.001/3).

### Cluster analysis of behavioral profiles

Changes in larval activity, startle response, habituation, excitability, and optomotor responses, as compared to the DMSO vehicle controls, were summarized in a ‘behavioral profile’. The profiles were created in MS Excel (template A) to examine 10 behaviors, as described in the section on ‘Data processing and outcome measures’. We then used MS Excel (template B), to calculate the average values of the treated groups (n = 48 larvae per drug) and subtract the average values of the DMSO vehicle controls (n = 844 larvae). The resulting numbers were color-coded by conditional formatting, to provide an overview of the effects of all 190 drugs on 10 parameters of behavior. In addition, the 190 behavioral profiles were compared to behavioral profiles of modulators of calcineurin signaling examined previously^26^. The numbers were imported in Cluster 3.0 for hierarchical cluster analysis, which can be used to group similar profiles. We used an ‘Eweight’ of 0.5 for all optomotor responses, without filtering or adjusting data, and used the Euclidian distance similarity metric with complete linkage. In contrast to correlation-based distance measures, the Euclidean distance takes the magnitude of changes into account. The clusters were shown in TreeView (version 1.1.6r4) using a spectrum from green (25% point decrease) to red (25% point increase).

## Supplementary Information

Supplementary Information 1.

Supplementary Information 2.

Supplementary Information 3.

Supplementary Information 4.

Supplementary Information 5.

Supplementary Information 6.

## Data Availability

Data, code, and materials used in the analysis are available in the supplementary information.
