# Supplementary material for: Novel use of FDA-approved drugs identified by cluster analysis of behavioral profiles
Source: Sci Rep. 2022 Apr 21;12:6120. doi: 10.1038/s41598-022-10133-y (PMC9023506; doi:10.1038/s41598-022-10133-y)

## Slide 1
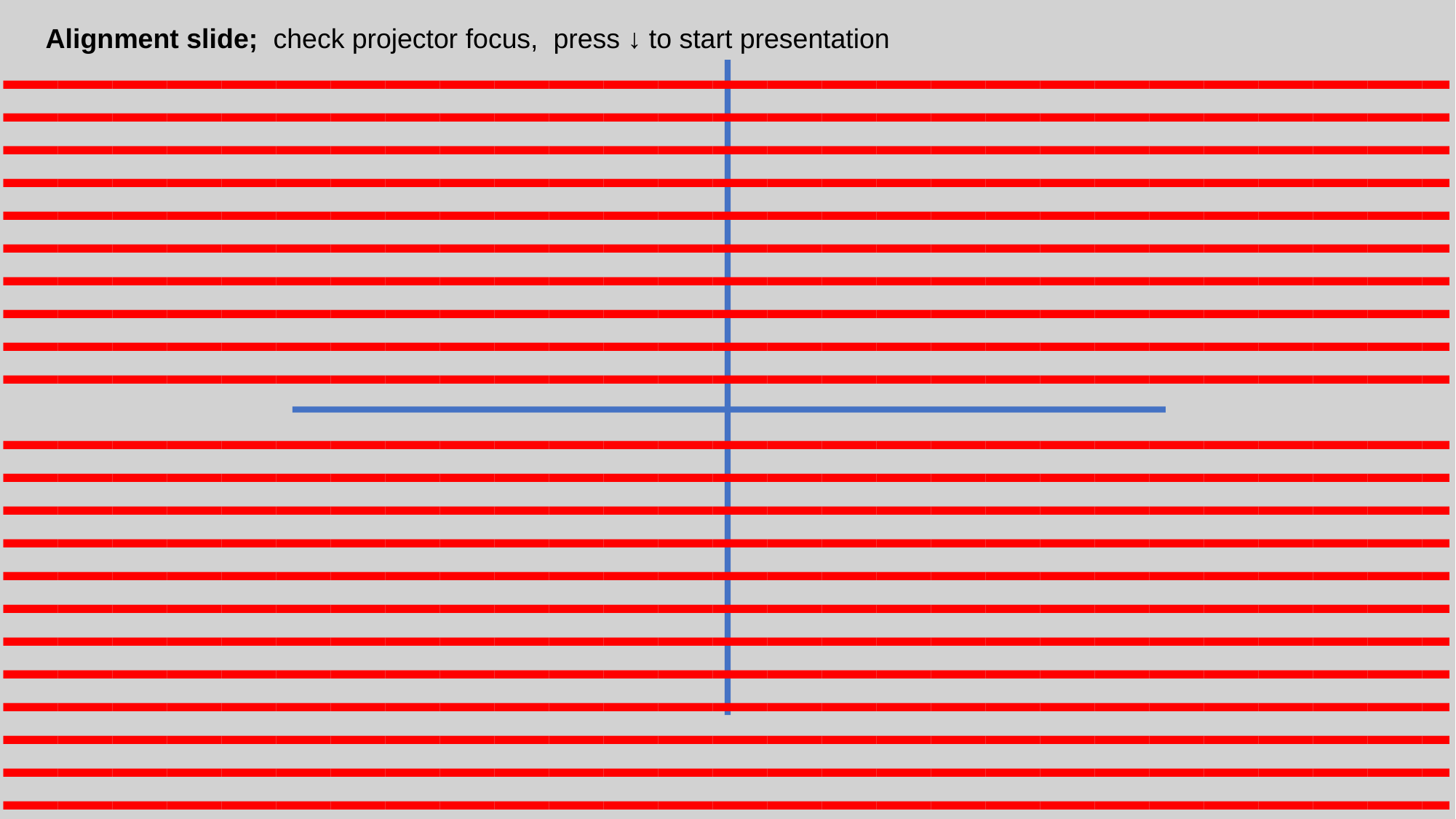

Alignment slide; check projector focus, press ↓ to start presentation
▬▬▬▬▬▬▬▬▬▬▬▬▬▬▬▬▬▬▬▬▬▬▬▬▬▬▬▬▬▬▬▬▬▬▬▬▬▬▬▬▬▬▬▬▬▬▬▬▬▬▬▬▬
▬▬▬▬▬▬▬▬▬▬▬▬▬▬▬▬▬▬▬▬▬▬▬▬▬▬▬▬▬▬▬▬▬▬▬▬▬▬▬▬▬▬▬▬▬▬▬▬▬▬▬▬▬
▬▬▬▬▬▬▬▬▬▬▬▬▬▬▬▬▬▬▬▬▬▬▬▬▬▬▬▬▬▬▬▬▬▬▬▬▬▬▬▬▬▬▬▬▬▬▬▬▬▬▬▬▬
▬▬▬▬▬▬▬▬▬▬▬▬▬▬▬▬▬▬▬▬▬▬▬▬▬▬▬▬▬▬▬▬▬▬▬▬▬▬▬▬▬▬▬▬▬▬▬▬▬▬▬▬▬
▬▬▬▬▬▬▬▬▬▬▬▬▬▬▬▬▬▬▬▬▬▬▬▬▬▬▬▬▬▬▬▬▬▬▬▬▬▬▬▬▬▬▬▬▬▬▬▬▬▬▬▬▬
▬▬▬▬▬▬▬▬▬▬▬▬▬▬▬▬▬▬▬▬▬▬▬▬▬▬▬▬▬▬▬▬▬▬▬▬▬▬▬▬▬▬▬▬▬▬▬▬▬▬▬▬▬
▬▬▬▬▬▬▬▬▬▬▬▬▬▬▬▬▬▬▬▬▬▬▬▬▬▬▬▬▬▬▬▬▬▬▬▬▬▬▬▬▬▬▬▬▬▬▬▬▬▬▬▬▬
▬▬▬▬▬▬▬▬▬▬▬▬▬▬▬▬▬▬▬▬▬▬▬▬▬▬▬▬▬▬▬▬▬▬▬▬▬▬▬▬▬▬▬▬▬▬▬▬▬▬▬▬▬
▬▬▬▬▬▬▬▬▬▬▬▬▬▬▬▬▬▬▬▬▬▬▬▬▬▬▬▬▬▬▬▬▬▬▬▬▬▬▬▬▬▬▬▬▬▬▬▬▬▬▬▬▬
▬▬▬▬▬▬▬▬▬▬▬▬▬▬▬▬▬▬▬▬▬▬▬▬▬▬▬▬▬▬▬▬▬▬▬▬▬▬▬▬▬▬▬▬▬▬▬▬▬▬▬▬▬
▬▬▬▬▬▬▬▬▬▬▬▬▬▬▬▬▬▬▬▬▬▬▬▬▬▬▬▬▬▬▬▬▬▬▬▬▬▬▬▬▬▬▬▬▬▬▬▬▬▬▬▬▬
▬▬▬▬▬▬▬▬▬▬▬▬▬▬▬▬▬▬▬▬▬▬▬▬▬▬▬▬▬▬▬▬▬▬▬▬▬▬▬▬▬▬▬▬▬▬▬▬▬▬▬▬▬
▬▬▬▬▬▬▬▬▬▬▬▬▬▬▬▬▬▬▬▬▬▬▬▬▬▬▬▬▬▬▬▬▬▬▬▬▬▬▬▬▬▬▬▬▬▬▬▬▬▬▬▬▬
▬▬▬▬▬▬▬▬▬▬▬▬▬▬▬▬▬▬▬▬▬▬▬▬▬▬▬▬▬▬▬▬▬▬▬▬▬▬▬▬▬▬▬▬▬▬▬▬▬▬▬▬▬
▬▬▬▬▬▬▬▬▬▬▬▬▬▬▬▬▬▬▬▬▬▬▬▬▬▬▬▬▬▬▬▬▬▬▬▬▬▬▬▬▬▬▬▬▬▬▬▬▬▬▬▬▬
▬▬▬▬▬▬▬▬▬▬▬▬▬▬▬▬▬▬▬▬▬▬▬▬▬▬▬▬▬▬▬▬▬▬▬▬▬▬▬▬▬▬▬▬▬▬▬▬▬▬▬▬▬
▬▬▬▬▬▬▬▬▬▬▬▬▬▬▬▬▬▬▬▬▬▬▬▬▬▬▬▬▬▬▬▬▬▬▬▬▬▬▬▬▬▬▬▬▬▬▬▬▬▬▬▬▬
▬▬▬▬▬▬▬▬▬▬▬▬▬▬▬▬▬▬▬▬▬▬▬▬▬▬▬▬▬▬▬▬▬▬▬▬▬▬▬▬▬▬▬▬▬▬▬▬▬▬▬▬▬
▬▬▬▬▬▬▬▬▬▬▬▬▬▬▬▬▬▬▬▬▬▬▬▬▬▬▬▬▬▬▬▬▬▬▬▬▬▬▬▬▬▬▬▬▬▬▬▬▬▬▬▬▬
▬▬▬▬▬▬▬▬▬▬▬▬▬▬▬▬▬▬▬▬▬▬▬▬▬▬▬▬▬▬▬▬▬▬▬▬▬▬▬▬▬▬▬▬▬▬▬▬▬▬▬▬▬
▬▬▬▬▬▬▬▬▬▬▬▬▬▬▬▬▬▬▬▬▬▬▬▬▬▬▬▬▬▬▬▬▬▬▬▬▬▬▬▬▬▬▬▬▬▬▬▬▬▬▬▬▬
▬▬▬▬▬▬▬▬▬▬▬▬▬▬▬▬▬▬▬▬▬▬▬▬▬▬▬▬▬▬▬▬▬▬▬▬▬▬▬▬▬▬▬▬▬▬▬▬▬▬▬▬▬

## Slide 2
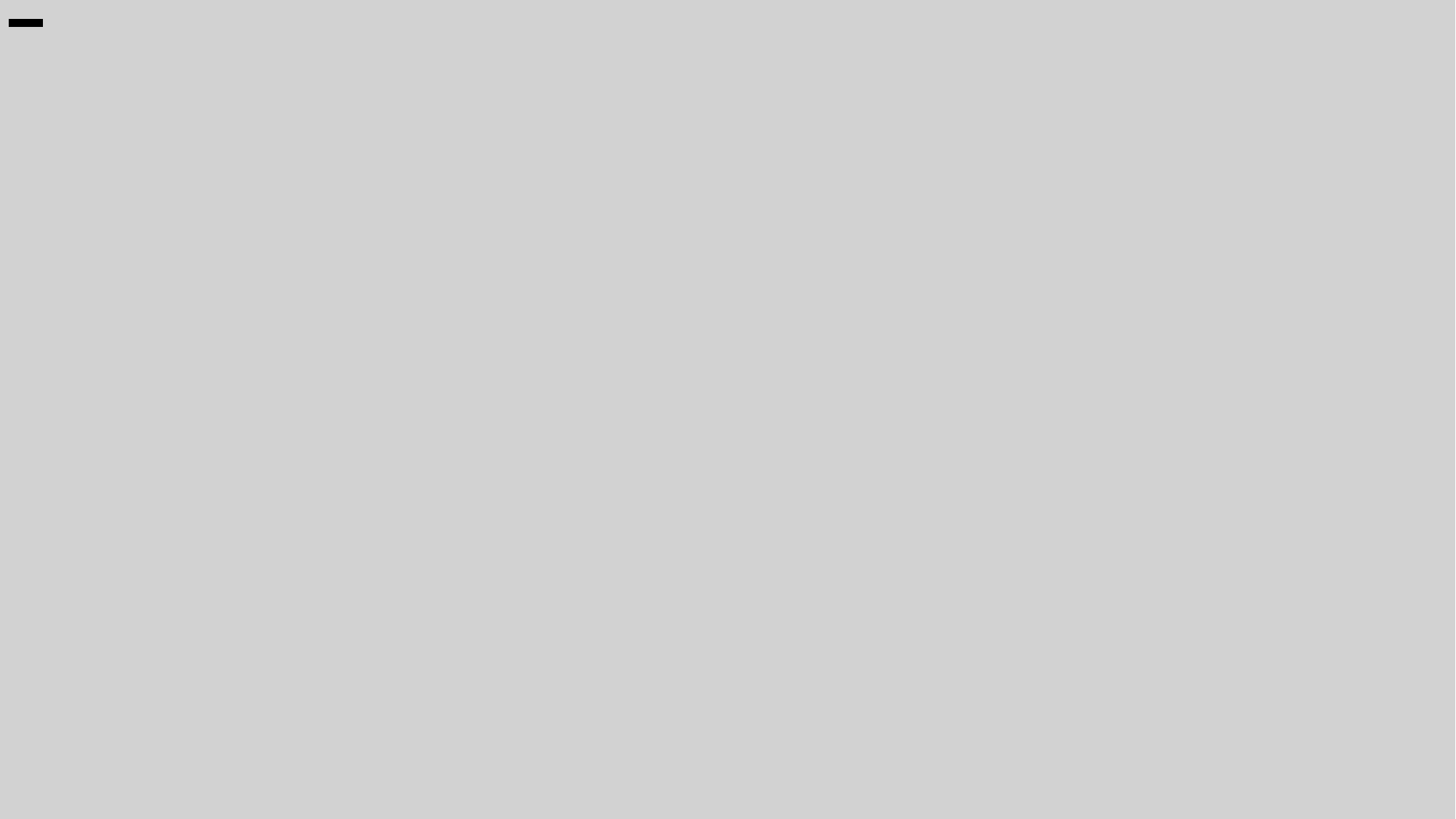

## Slide 3
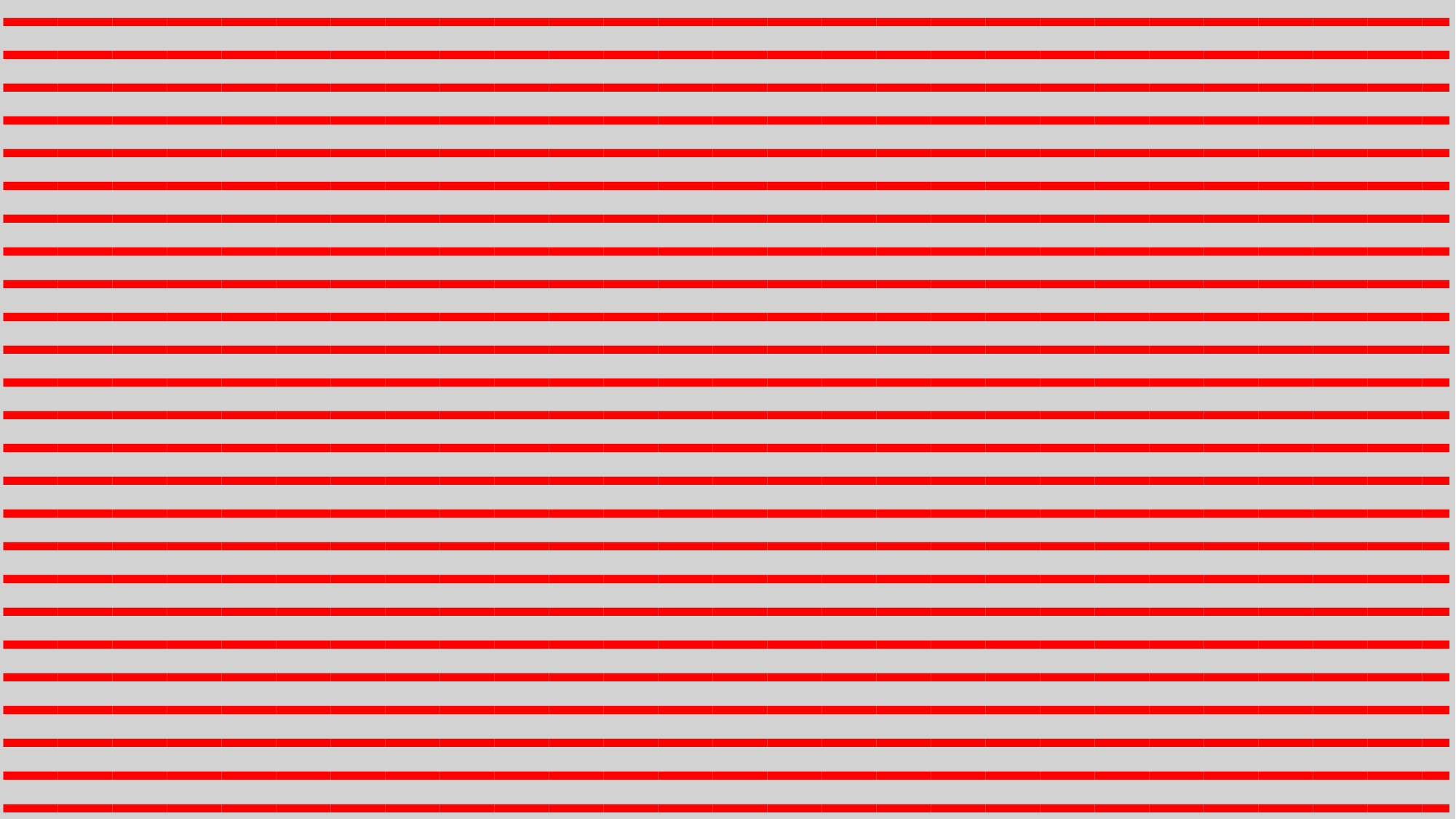

▬▬▬▬▬▬▬▬▬▬▬▬▬▬▬▬▬▬▬▬▬▬▬▬▬▬▬▬▬▬▬▬▬▬▬▬▬▬▬▬▬▬▬▬▬▬▬▬▬▬▬▬▬
▬▬▬▬▬▬▬▬▬▬▬▬▬▬▬▬▬▬▬▬▬▬▬▬▬▬▬▬▬▬▬▬▬▬▬▬▬▬▬▬▬▬▬▬▬▬▬▬▬▬▬▬▬
▬▬▬▬▬▬▬▬▬▬▬▬▬▬▬▬▬▬▬▬▬▬▬▬▬▬▬▬▬▬▬▬▬▬▬▬▬▬▬▬▬▬▬▬▬▬▬▬▬▬▬▬▬
▬▬▬▬▬▬▬▬▬▬▬▬▬▬▬▬▬▬▬▬▬▬▬▬▬▬▬▬▬▬▬▬▬▬▬▬▬▬▬▬▬▬▬▬▬▬▬▬▬▬▬▬▬
▬▬▬▬▬▬▬▬▬▬▬▬▬▬▬▬▬▬▬▬▬▬▬▬▬▬▬▬▬▬▬▬▬▬▬▬▬▬▬▬▬▬▬▬▬▬▬▬▬▬▬▬▬
▬▬▬▬▬▬▬▬▬▬▬▬▬▬▬▬▬▬▬▬▬▬▬▬▬▬▬▬▬▬▬▬▬▬▬▬▬▬▬▬▬▬▬▬▬▬▬▬▬▬▬▬▬
▬▬▬▬▬▬▬▬▬▬▬▬▬▬▬▬▬▬▬▬▬▬▬▬▬▬▬▬▬▬▬▬▬▬▬▬▬▬▬▬▬▬▬▬▬▬▬▬▬▬▬▬▬
▬▬▬▬▬▬▬▬▬▬▬▬▬▬▬▬▬▬▬▬▬▬▬▬▬▬▬▬▬▬▬▬▬▬▬▬▬▬▬▬▬▬▬▬▬▬▬▬▬▬▬▬▬
▬▬▬▬▬▬▬▬▬▬▬▬▬▬▬▬▬▬▬▬▬▬▬▬▬▬▬▬▬▬▬▬▬▬▬▬▬▬▬▬▬▬▬▬▬▬▬▬▬▬▬▬▬
▬▬▬▬▬▬▬▬▬▬▬▬▬▬▬▬▬▬▬▬▬▬▬▬▬▬▬▬▬▬▬▬▬▬▬▬▬▬▬▬▬▬▬▬▬▬▬▬▬▬▬▬▬
▬▬▬▬▬▬▬▬▬▬▬▬▬▬▬▬▬▬▬▬▬▬▬▬▬▬▬▬▬▬▬▬▬▬▬▬▬▬▬▬▬▬▬▬▬▬▬▬▬▬▬▬▬
▬▬▬▬▬▬▬▬▬▬▬▬▬▬▬▬▬▬▬▬▬▬▬▬▬▬▬▬▬▬▬▬▬▬▬▬▬▬▬▬▬▬▬▬▬▬▬▬▬▬▬▬▬
▬▬▬▬▬▬▬▬▬▬▬▬▬▬▬▬▬▬▬▬▬▬▬▬▬▬▬▬▬▬▬▬▬▬▬▬▬▬▬▬▬▬▬▬▬▬▬▬▬▬▬▬▬
▬▬▬▬▬▬▬▬▬▬▬▬▬▬▬▬▬▬▬▬▬▬▬▬▬▬▬▬▬▬▬▬▬▬▬▬▬▬▬▬▬▬▬▬▬▬▬▬▬▬▬▬▬
▬▬▬▬▬▬▬▬▬▬▬▬▬▬▬▬▬▬▬▬▬▬▬▬▬▬▬▬▬▬▬▬▬▬▬▬▬▬▬▬▬▬▬▬▬▬▬▬▬▬▬▬▬
▬▬▬▬▬▬▬▬▬▬▬▬▬▬▬▬▬▬▬▬▬▬▬▬▬▬▬▬▬▬▬▬▬▬▬▬▬▬▬▬▬▬▬▬▬▬▬▬▬▬▬▬▬
▬▬▬▬▬▬▬▬▬▬▬▬▬▬▬▬▬▬▬▬▬▬▬▬▬▬▬▬▬▬▬▬▬▬▬▬▬▬▬▬▬▬▬▬▬▬▬▬▬▬▬▬▬
▬▬▬▬▬▬▬▬▬▬▬▬▬▬▬▬▬▬▬▬▬▬▬▬▬▬▬▬▬▬▬▬▬▬▬▬▬▬▬▬▬▬▬▬▬▬▬▬▬▬▬▬▬
▬▬▬▬▬▬▬▬▬▬▬▬▬▬▬▬▬▬▬▬▬▬▬▬▬▬▬▬▬▬▬▬▬▬▬▬▬▬▬▬▬▬▬▬▬▬▬▬▬▬▬▬▬
▬▬▬▬▬▬▬▬▬▬▬▬▬▬▬▬▬▬▬▬▬▬▬▬▬▬▬▬▬▬▬▬▬▬▬▬▬▬▬▬▬▬▬▬▬▬▬▬▬▬▬▬▬
▬▬▬▬▬▬▬▬▬▬▬▬▬▬▬▬▬▬▬▬▬▬▬▬▬▬▬▬▬▬▬▬▬▬▬▬▬▬▬▬▬▬▬▬▬▬▬▬▬▬▬▬▬
▬▬▬▬▬▬▬▬▬▬▬▬▬▬▬▬▬▬▬▬▬▬▬▬▬▬▬▬▬▬▬▬▬▬▬▬▬▬▬▬▬▬▬▬▬▬▬▬▬▬▬▬▬
▬▬▬▬▬▬▬▬▬▬▬▬▬▬▬▬▬▬▬▬▬▬▬▬▬▬▬▬▬▬▬▬▬▬▬▬▬▬▬▬▬▬▬▬▬▬▬▬▬▬▬▬▬
▬▬▬▬▬▬▬▬▬▬▬▬▬▬▬▬▬▬▬▬▬▬▬▬▬▬▬▬▬▬▬▬▬▬▬▬▬▬▬▬▬▬▬▬▬▬▬▬▬▬▬▬▬
▬▬▬▬▬▬▬▬▬▬▬▬▬▬▬▬▬▬▬▬▬▬▬▬▬▬▬▬▬▬▬▬▬▬▬▬▬▬▬▬▬▬▬▬▬▬▬▬▬▬▬▬▬
▬▬▬▬▬▬▬▬▬▬▬▬▬▬▬▬▬▬▬▬▬▬▬▬▬▬▬▬▬▬▬▬▬▬▬▬▬▬▬▬▬▬▬▬▬▬▬▬▬▬▬▬▬
▬▬▬▬▬▬▬▬▬▬▬▬▬▬▬▬▬▬▬▬▬▬▬▬▬▬▬▬▬▬▬▬▬▬▬▬▬▬▬▬▬▬▬▬▬▬▬▬▬▬▬▬▬
▬▬▬▬▬▬▬▬▬▬▬▬▬▬▬▬▬▬▬▬▬▬▬▬▬▬▬▬▬▬▬▬▬▬▬▬▬▬▬▬▬▬▬▬▬▬▬▬▬▬▬▬▬
▬▬▬▬▬▬▬▬▬▬▬▬▬▬▬▬▬▬▬▬▬▬▬▬▬▬▬▬▬▬▬▬▬▬▬▬▬▬▬▬▬▬▬▬▬▬▬▬▬▬▬▬▬

## Slide 4
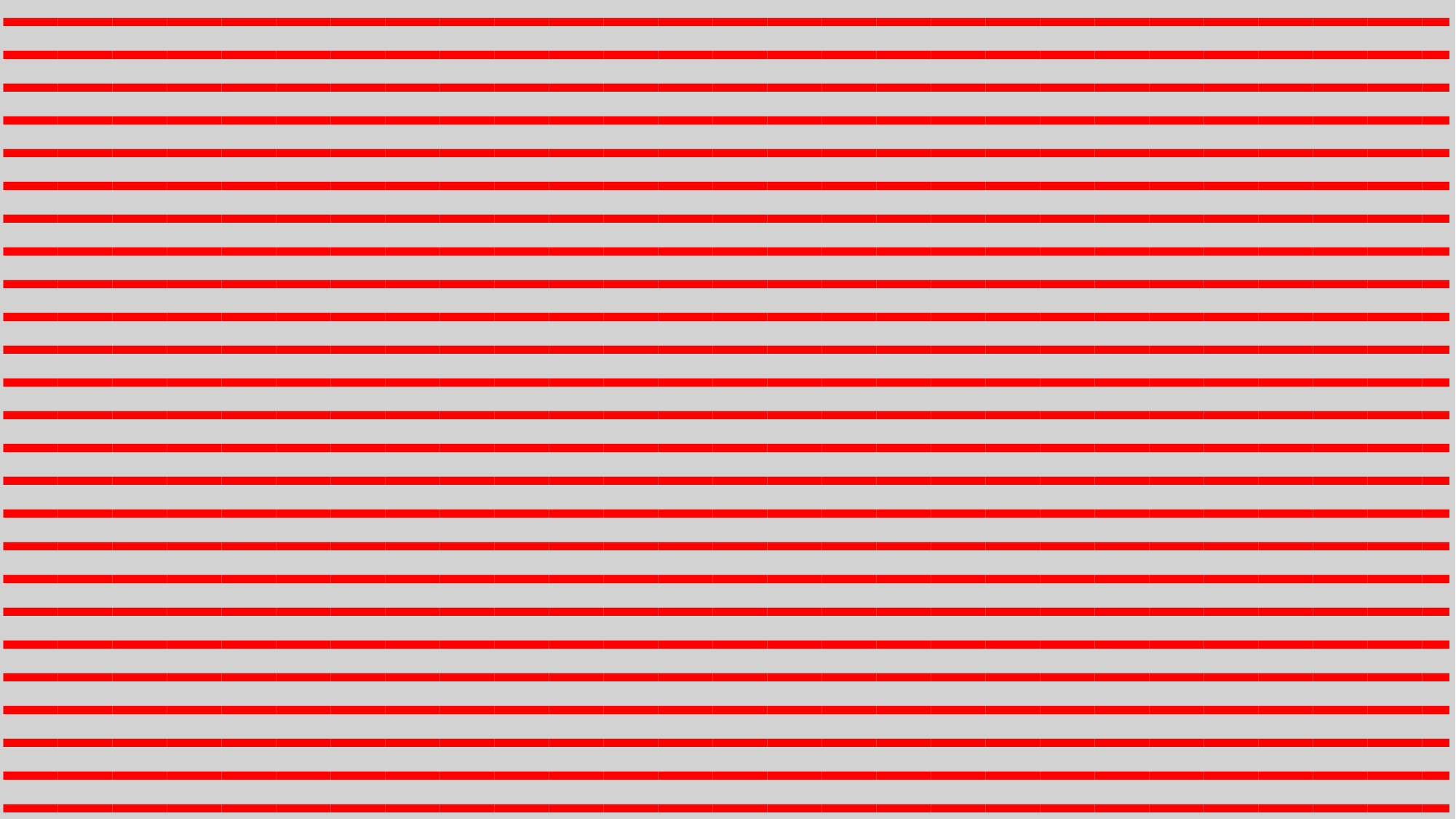

▬▬▬▬▬▬▬▬▬▬▬▬▬▬▬▬▬▬▬▬▬▬▬▬▬▬▬▬▬▬▬▬▬▬▬▬▬▬▬▬▬▬▬▬▬▬▬▬▬▬▬▬▬
▬▬▬▬▬▬▬▬▬▬▬▬▬▬▬▬▬▬▬▬▬▬▬▬▬▬▬▬▬▬▬▬▬▬▬▬▬▬▬▬▬▬▬▬▬▬▬▬▬▬▬▬▬
▬▬▬▬▬▬▬▬▬▬▬▬▬▬▬▬▬▬▬▬▬▬▬▬▬▬▬▬▬▬▬▬▬▬▬▬▬▬▬▬▬▬▬▬▬▬▬▬▬▬▬▬▬
▬▬▬▬▬▬▬▬▬▬▬▬▬▬▬▬▬▬▬▬▬▬▬▬▬▬▬▬▬▬▬▬▬▬▬▬▬▬▬▬▬▬▬▬▬▬▬▬▬▬▬▬▬
▬▬▬▬▬▬▬▬▬▬▬▬▬▬▬▬▬▬▬▬▬▬▬▬▬▬▬▬▬▬▬▬▬▬▬▬▬▬▬▬▬▬▬▬▬▬▬▬▬▬▬▬▬
▬▬▬▬▬▬▬▬▬▬▬▬▬▬▬▬▬▬▬▬▬▬▬▬▬▬▬▬▬▬▬▬▬▬▬▬▬▬▬▬▬▬▬▬▬▬▬▬▬▬▬▬▬
▬▬▬▬▬▬▬▬▬▬▬▬▬▬▬▬▬▬▬▬▬▬▬▬▬▬▬▬▬▬▬▬▬▬▬▬▬▬▬▬▬▬▬▬▬▬▬▬▬▬▬▬▬
▬▬▬▬▬▬▬▬▬▬▬▬▬▬▬▬▬▬▬▬▬▬▬▬▬▬▬▬▬▬▬▬▬▬▬▬▬▬▬▬▬▬▬▬▬▬▬▬▬▬▬▬▬
▬▬▬▬▬▬▬▬▬▬▬▬▬▬▬▬▬▬▬▬▬▬▬▬▬▬▬▬▬▬▬▬▬▬▬▬▬▬▬▬▬▬▬▬▬▬▬▬▬▬▬▬▬
▬▬▬▬▬▬▬▬▬▬▬▬▬▬▬▬▬▬▬▬▬▬▬▬▬▬▬▬▬▬▬▬▬▬▬▬▬▬▬▬▬▬▬▬▬▬▬▬▬▬▬▬▬
▬▬▬▬▬▬▬▬▬▬▬▬▬▬▬▬▬▬▬▬▬▬▬▬▬▬▬▬▬▬▬▬▬▬▬▬▬▬▬▬▬▬▬▬▬▬▬▬▬▬▬▬▬
▬▬▬▬▬▬▬▬▬▬▬▬▬▬▬▬▬▬▬▬▬▬▬▬▬▬▬▬▬▬▬▬▬▬▬▬▬▬▬▬▬▬▬▬▬▬▬▬▬▬▬▬▬
▬▬▬▬▬▬▬▬▬▬▬▬▬▬▬▬▬▬▬▬▬▬▬▬▬▬▬▬▬▬▬▬▬▬▬▬▬▬▬▬▬▬▬▬▬▬▬▬▬▬▬▬▬
▬▬▬▬▬▬▬▬▬▬▬▬▬▬▬▬▬▬▬▬▬▬▬▬▬▬▬▬▬▬▬▬▬▬▬▬▬▬▬▬▬▬▬▬▬▬▬▬▬▬▬▬▬
▬▬▬▬▬▬▬▬▬▬▬▬▬▬▬▬▬▬▬▬▬▬▬▬▬▬▬▬▬▬▬▬▬▬▬▬▬▬▬▬▬▬▬▬▬▬▬▬▬▬▬▬▬
▬▬▬▬▬▬▬▬▬▬▬▬▬▬▬▬▬▬▬▬▬▬▬▬▬▬▬▬▬▬▬▬▬▬▬▬▬▬▬▬▬▬▬▬▬▬▬▬▬▬▬▬▬
▬▬▬▬▬▬▬▬▬▬▬▬▬▬▬▬▬▬▬▬▬▬▬▬▬▬▬▬▬▬▬▬▬▬▬▬▬▬▬▬▬▬▬▬▬▬▬▬▬▬▬▬▬
▬▬▬▬▬▬▬▬▬▬▬▬▬▬▬▬▬▬▬▬▬▬▬▬▬▬▬▬▬▬▬▬▬▬▬▬▬▬▬▬▬▬▬▬▬▬▬▬▬▬▬▬▬
▬▬▬▬▬▬▬▬▬▬▬▬▬▬▬▬▬▬▬▬▬▬▬▬▬▬▬▬▬▬▬▬▬▬▬▬▬▬▬▬▬▬▬▬▬▬▬▬▬▬▬▬▬
▬▬▬▬▬▬▬▬▬▬▬▬▬▬▬▬▬▬▬▬▬▬▬▬▬▬▬▬▬▬▬▬▬▬▬▬▬▬▬▬▬▬▬▬▬▬▬▬▬▬▬▬▬
▬▬▬▬▬▬▬▬▬▬▬▬▬▬▬▬▬▬▬▬▬▬▬▬▬▬▬▬▬▬▬▬▬▬▬▬▬▬▬▬▬▬▬▬▬▬▬▬▬▬▬▬▬
▬▬▬▬▬▬▬▬▬▬▬▬▬▬▬▬▬▬▬▬▬▬▬▬▬▬▬▬▬▬▬▬▬▬▬▬▬▬▬▬▬▬▬▬▬▬▬▬▬▬▬▬▬
▬▬▬▬▬▬▬▬▬▬▬▬▬▬▬▬▬▬▬▬▬▬▬▬▬▬▬▬▬▬▬▬▬▬▬▬▬▬▬▬▬▬▬▬▬▬▬▬▬▬▬▬▬
▬▬▬▬▬▬▬▬▬▬▬▬▬▬▬▬▬▬▬▬▬▬▬▬▬▬▬▬▬▬▬▬▬▬▬▬▬▬▬▬▬▬▬▬▬▬▬▬▬▬▬▬▬
▬▬▬▬▬▬▬▬▬▬▬▬▬▬▬▬▬▬▬▬▬▬▬▬▬▬▬▬▬▬▬▬▬▬▬▬▬▬▬▬▬▬▬▬▬▬▬▬▬▬▬▬▬
▬▬▬▬▬▬▬▬▬▬▬▬▬▬▬▬▬▬▬▬▬▬▬▬▬▬▬▬▬▬▬▬▬▬▬▬▬▬▬▬▬▬▬▬▬▬▬▬▬▬▬▬▬
▬▬▬▬▬▬▬▬▬▬▬▬▬▬▬▬▬▬▬▬▬▬▬▬▬▬▬▬▬▬▬▬▬▬▬▬▬▬▬▬▬▬▬▬▬▬▬▬▬▬▬▬▬
▬▬▬▬▬▬▬▬▬▬▬▬▬▬▬▬▬▬▬▬▬▬▬▬▬▬▬▬▬▬▬▬▬▬▬▬▬▬▬▬▬▬▬▬▬▬▬▬▬▬▬▬▬
▬▬▬▬▬▬▬▬▬▬▬▬▬▬▬▬▬▬▬▬▬▬▬▬▬▬▬▬▬▬▬▬▬▬▬▬▬▬▬▬▬▬▬▬▬▬▬▬▬▬▬▬▬

## Slide 5
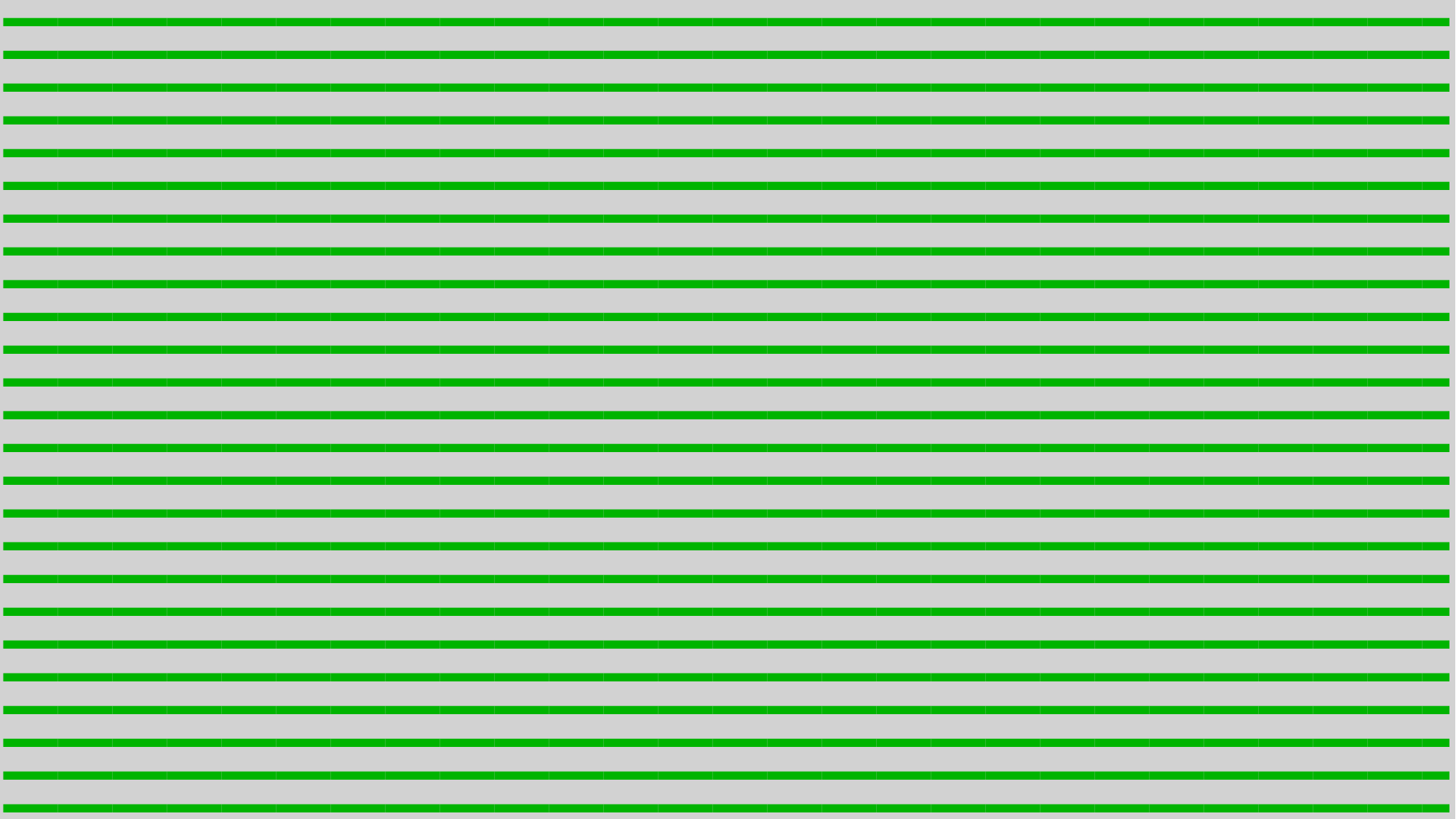

▬▬▬▬▬▬▬▬▬▬▬▬▬▬▬▬▬▬▬▬▬▬▬▬▬▬▬▬▬▬▬▬▬▬▬▬▬▬▬▬▬▬▬▬▬▬▬▬▬▬▬▬▬
▬▬▬▬▬▬▬▬▬▬▬▬▬▬▬▬▬▬▬▬▬▬▬▬▬▬▬▬▬▬▬▬▬▬▬▬▬▬▬▬▬▬▬▬▬▬▬▬▬▬▬▬▬
▬▬▬▬▬▬▬▬▬▬▬▬▬▬▬▬▬▬▬▬▬▬▬▬▬▬▬▬▬▬▬▬▬▬▬▬▬▬▬▬▬▬▬▬▬▬▬▬▬▬▬▬▬
▬▬▬▬▬▬▬▬▬▬▬▬▬▬▬▬▬▬▬▬▬▬▬▬▬▬▬▬▬▬▬▬▬▬▬▬▬▬▬▬▬▬▬▬▬▬▬▬▬▬▬▬▬
▬▬▬▬▬▬▬▬▬▬▬▬▬▬▬▬▬▬▬▬▬▬▬▬▬▬▬▬▬▬▬▬▬▬▬▬▬▬▬▬▬▬▬▬▬▬▬▬▬▬▬▬▬
▬▬▬▬▬▬▬▬▬▬▬▬▬▬▬▬▬▬▬▬▬▬▬▬▬▬▬▬▬▬▬▬▬▬▬▬▬▬▬▬▬▬▬▬▬▬▬▬▬▬▬▬▬
▬▬▬▬▬▬▬▬▬▬▬▬▬▬▬▬▬▬▬▬▬▬▬▬▬▬▬▬▬▬▬▬▬▬▬▬▬▬▬▬▬▬▬▬▬▬▬▬▬▬▬▬▬
▬▬▬▬▬▬▬▬▬▬▬▬▬▬▬▬▬▬▬▬▬▬▬▬▬▬▬▬▬▬▬▬▬▬▬▬▬▬▬▬▬▬▬▬▬▬▬▬▬▬▬▬▬
▬▬▬▬▬▬▬▬▬▬▬▬▬▬▬▬▬▬▬▬▬▬▬▬▬▬▬▬▬▬▬▬▬▬▬▬▬▬▬▬▬▬▬▬▬▬▬▬▬▬▬▬▬
▬▬▬▬▬▬▬▬▬▬▬▬▬▬▬▬▬▬▬▬▬▬▬▬▬▬▬▬▬▬▬▬▬▬▬▬▬▬▬▬▬▬▬▬▬▬▬▬▬▬▬▬▬
▬▬▬▬▬▬▬▬▬▬▬▬▬▬▬▬▬▬▬▬▬▬▬▬▬▬▬▬▬▬▬▬▬▬▬▬▬▬▬▬▬▬▬▬▬▬▬▬▬▬▬▬▬
▬▬▬▬▬▬▬▬▬▬▬▬▬▬▬▬▬▬▬▬▬▬▬▬▬▬▬▬▬▬▬▬▬▬▬▬▬▬▬▬▬▬▬▬▬▬▬▬▬▬▬▬▬
▬▬▬▬▬▬▬▬▬▬▬▬▬▬▬▬▬▬▬▬▬▬▬▬▬▬▬▬▬▬▬▬▬▬▬▬▬▬▬▬▬▬▬▬▬▬▬▬▬▬▬▬▬
▬▬▬▬▬▬▬▬▬▬▬▬▬▬▬▬▬▬▬▬▬▬▬▬▬▬▬▬▬▬▬▬▬▬▬▬▬▬▬▬▬▬▬▬▬▬▬▬▬▬▬▬▬
▬▬▬▬▬▬▬▬▬▬▬▬▬▬▬▬▬▬▬▬▬▬▬▬▬▬▬▬▬▬▬▬▬▬▬▬▬▬▬▬▬▬▬▬▬▬▬▬▬▬▬▬▬
▬▬▬▬▬▬▬▬▬▬▬▬▬▬▬▬▬▬▬▬▬▬▬▬▬▬▬▬▬▬▬▬▬▬▬▬▬▬▬▬▬▬▬▬▬▬▬▬▬▬▬▬▬
▬▬▬▬▬▬▬▬▬▬▬▬▬▬▬▬▬▬▬▬▬▬▬▬▬▬▬▬▬▬▬▬▬▬▬▬▬▬▬▬▬▬▬▬▬▬▬▬▬▬▬▬▬
▬▬▬▬▬▬▬▬▬▬▬▬▬▬▬▬▬▬▬▬▬▬▬▬▬▬▬▬▬▬▬▬▬▬▬▬▬▬▬▬▬▬▬▬▬▬▬▬▬▬▬▬▬
▬▬▬▬▬▬▬▬▬▬▬▬▬▬▬▬▬▬▬▬▬▬▬▬▬▬▬▬▬▬▬▬▬▬▬▬▬▬▬▬▬▬▬▬▬▬▬▬▬▬▬▬▬
▬▬▬▬▬▬▬▬▬▬▬▬▬▬▬▬▬▬▬▬▬▬▬▬▬▬▬▬▬▬▬▬▬▬▬▬▬▬▬▬▬▬▬▬▬▬▬▬▬▬▬▬▬
▬▬▬▬▬▬▬▬▬▬▬▬▬▬▬▬▬▬▬▬▬▬▬▬▬▬▬▬▬▬▬▬▬▬▬▬▬▬▬▬▬▬▬▬▬▬▬▬▬▬▬▬▬
▬▬▬▬▬▬▬▬▬▬▬▬▬▬▬▬▬▬▬▬▬▬▬▬▬▬▬▬▬▬▬▬▬▬▬▬▬▬▬▬▬▬▬▬▬▬▬▬▬▬▬▬▬
▬▬▬▬▬▬▬▬▬▬▬▬▬▬▬▬▬▬▬▬▬▬▬▬▬▬▬▬▬▬▬▬▬▬▬▬▬▬▬▬▬▬▬▬▬▬▬▬▬▬▬▬▬
▬▬▬▬▬▬▬▬▬▬▬▬▬▬▬▬▬▬▬▬▬▬▬▬▬▬▬▬▬▬▬▬▬▬▬▬▬▬▬▬▬▬▬▬▬▬▬▬▬▬▬▬▬
▬▬▬▬▬▬▬▬▬▬▬▬▬▬▬▬▬▬▬▬▬▬▬▬▬▬▬▬▬▬▬▬▬▬▬▬▬▬▬▬▬▬▬▬▬▬▬▬▬▬▬▬▬
▬▬▬▬▬▬▬▬▬▬▬▬▬▬▬▬▬▬▬▬▬▬▬▬▬▬▬▬▬▬▬▬▬▬▬▬▬▬▬▬▬▬▬▬▬▬▬▬▬▬▬▬▬
▬▬▬▬▬▬▬▬▬▬▬▬▬▬▬▬▬▬▬▬▬▬▬▬▬▬▬▬▬▬▬▬▬▬▬▬▬▬▬▬▬▬▬▬▬▬▬▬▬▬▬▬▬
▬▬▬▬▬▬▬▬▬▬▬▬▬▬▬▬▬▬▬▬▬▬▬▬▬▬▬▬▬▬▬▬▬▬▬▬▬▬▬▬▬▬▬▬▬▬▬▬▬▬▬▬▬
▬▬▬▬▬▬▬▬▬▬▬▬▬▬▬▬▬▬▬▬▬▬▬▬▬▬▬▬▬▬▬▬▬▬▬▬▬▬▬▬▬▬▬▬▬▬▬▬▬▬▬▬▬

## Slide 6
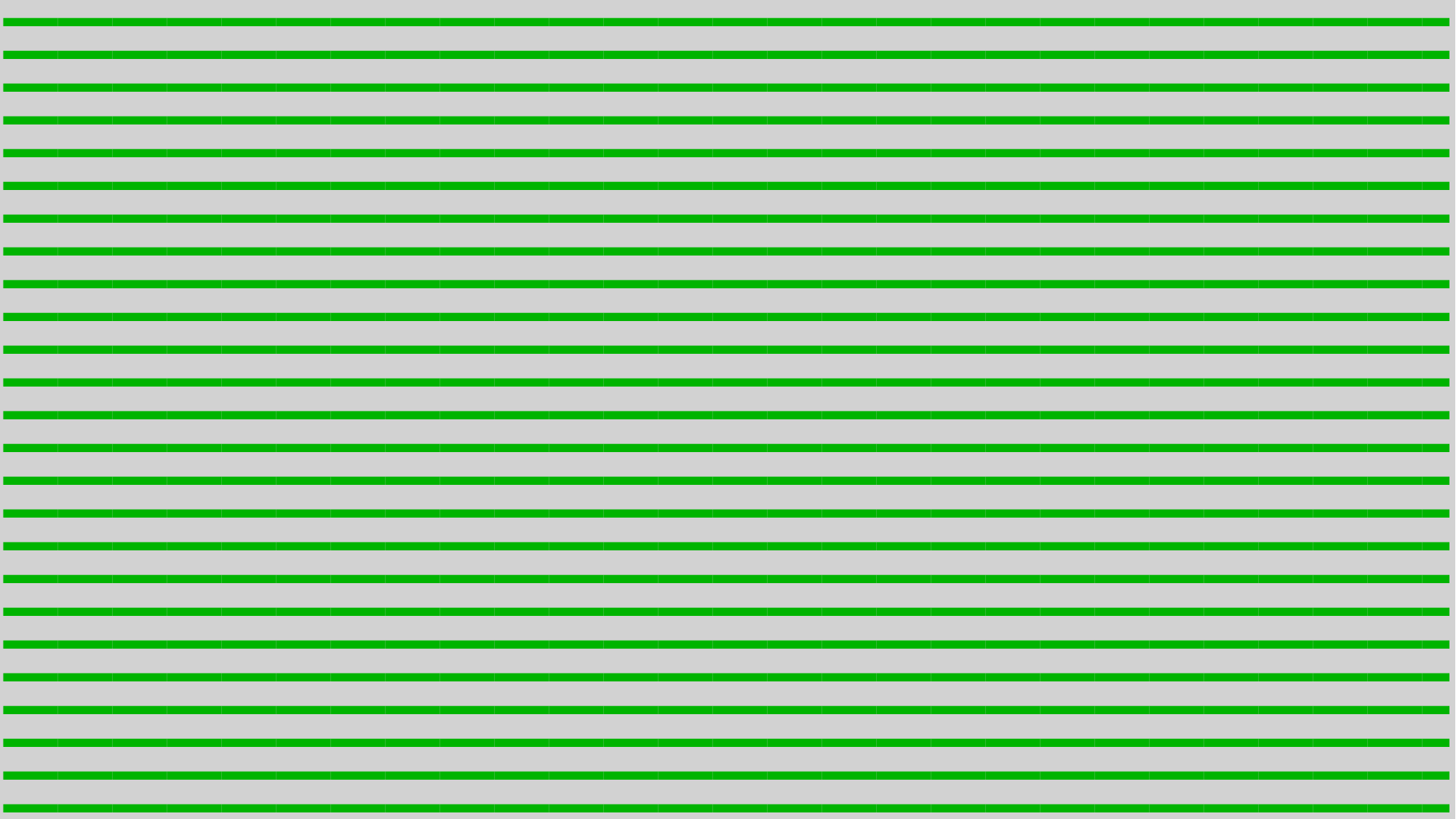

▬▬▬▬▬▬▬▬▬▬▬▬▬▬▬▬▬▬▬▬▬▬▬▬▬▬▬▬▬▬▬▬▬▬▬▬▬▬▬▬▬▬▬▬▬▬▬▬▬▬▬▬▬
▬▬▬▬▬▬▬▬▬▬▬▬▬▬▬▬▬▬▬▬▬▬▬▬▬▬▬▬▬▬▬▬▬▬▬▬▬▬▬▬▬▬▬▬▬▬▬▬▬▬▬▬▬
▬▬▬▬▬▬▬▬▬▬▬▬▬▬▬▬▬▬▬▬▬▬▬▬▬▬▬▬▬▬▬▬▬▬▬▬▬▬▬▬▬▬▬▬▬▬▬▬▬▬▬▬▬
▬▬▬▬▬▬▬▬▬▬▬▬▬▬▬▬▬▬▬▬▬▬▬▬▬▬▬▬▬▬▬▬▬▬▬▬▬▬▬▬▬▬▬▬▬▬▬▬▬▬▬▬▬
▬▬▬▬▬▬▬▬▬▬▬▬▬▬▬▬▬▬▬▬▬▬▬▬▬▬▬▬▬▬▬▬▬▬▬▬▬▬▬▬▬▬▬▬▬▬▬▬▬▬▬▬▬
▬▬▬▬▬▬▬▬▬▬▬▬▬▬▬▬▬▬▬▬▬▬▬▬▬▬▬▬▬▬▬▬▬▬▬▬▬▬▬▬▬▬▬▬▬▬▬▬▬▬▬▬▬
▬▬▬▬▬▬▬▬▬▬▬▬▬▬▬▬▬▬▬▬▬▬▬▬▬▬▬▬▬▬▬▬▬▬▬▬▬▬▬▬▬▬▬▬▬▬▬▬▬▬▬▬▬
▬▬▬▬▬▬▬▬▬▬▬▬▬▬▬▬▬▬▬▬▬▬▬▬▬▬▬▬▬▬▬▬▬▬▬▬▬▬▬▬▬▬▬▬▬▬▬▬▬▬▬▬▬
▬▬▬▬▬▬▬▬▬▬▬▬▬▬▬▬▬▬▬▬▬▬▬▬▬▬▬▬▬▬▬▬▬▬▬▬▬▬▬▬▬▬▬▬▬▬▬▬▬▬▬▬▬
▬▬▬▬▬▬▬▬▬▬▬▬▬▬▬▬▬▬▬▬▬▬▬▬▬▬▬▬▬▬▬▬▬▬▬▬▬▬▬▬▬▬▬▬▬▬▬▬▬▬▬▬▬
▬▬▬▬▬▬▬▬▬▬▬▬▬▬▬▬▬▬▬▬▬▬▬▬▬▬▬▬▬▬▬▬▬▬▬▬▬▬▬▬▬▬▬▬▬▬▬▬▬▬▬▬▬
▬▬▬▬▬▬▬▬▬▬▬▬▬▬▬▬▬▬▬▬▬▬▬▬▬▬▬▬▬▬▬▬▬▬▬▬▬▬▬▬▬▬▬▬▬▬▬▬▬▬▬▬▬
▬▬▬▬▬▬▬▬▬▬▬▬▬▬▬▬▬▬▬▬▬▬▬▬▬▬▬▬▬▬▬▬▬▬▬▬▬▬▬▬▬▬▬▬▬▬▬▬▬▬▬▬▬
▬▬▬▬▬▬▬▬▬▬▬▬▬▬▬▬▬▬▬▬▬▬▬▬▬▬▬▬▬▬▬▬▬▬▬▬▬▬▬▬▬▬▬▬▬▬▬▬▬▬▬▬▬
▬▬▬▬▬▬▬▬▬▬▬▬▬▬▬▬▬▬▬▬▬▬▬▬▬▬▬▬▬▬▬▬▬▬▬▬▬▬▬▬▬▬▬▬▬▬▬▬▬▬▬▬▬
▬▬▬▬▬▬▬▬▬▬▬▬▬▬▬▬▬▬▬▬▬▬▬▬▬▬▬▬▬▬▬▬▬▬▬▬▬▬▬▬▬▬▬▬▬▬▬▬▬▬▬▬▬
▬▬▬▬▬▬▬▬▬▬▬▬▬▬▬▬▬▬▬▬▬▬▬▬▬▬▬▬▬▬▬▬▬▬▬▬▬▬▬▬▬▬▬▬▬▬▬▬▬▬▬▬▬
▬▬▬▬▬▬▬▬▬▬▬▬▬▬▬▬▬▬▬▬▬▬▬▬▬▬▬▬▬▬▬▬▬▬▬▬▬▬▬▬▬▬▬▬▬▬▬▬▬▬▬▬▬
▬▬▬▬▬▬▬▬▬▬▬▬▬▬▬▬▬▬▬▬▬▬▬▬▬▬▬▬▬▬▬▬▬▬▬▬▬▬▬▬▬▬▬▬▬▬▬▬▬▬▬▬▬
▬▬▬▬▬▬▬▬▬▬▬▬▬▬▬▬▬▬▬▬▬▬▬▬▬▬▬▬▬▬▬▬▬▬▬▬▬▬▬▬▬▬▬▬▬▬▬▬▬▬▬▬▬
▬▬▬▬▬▬▬▬▬▬▬▬▬▬▬▬▬▬▬▬▬▬▬▬▬▬▬▬▬▬▬▬▬▬▬▬▬▬▬▬▬▬▬▬▬▬▬▬▬▬▬▬▬
▬▬▬▬▬▬▬▬▬▬▬▬▬▬▬▬▬▬▬▬▬▬▬▬▬▬▬▬▬▬▬▬▬▬▬▬▬▬▬▬▬▬▬▬▬▬▬▬▬▬▬▬▬
▬▬▬▬▬▬▬▬▬▬▬▬▬▬▬▬▬▬▬▬▬▬▬▬▬▬▬▬▬▬▬▬▬▬▬▬▬▬▬▬▬▬▬▬▬▬▬▬▬▬▬▬▬
▬▬▬▬▬▬▬▬▬▬▬▬▬▬▬▬▬▬▬▬▬▬▬▬▬▬▬▬▬▬▬▬▬▬▬▬▬▬▬▬▬▬▬▬▬▬▬▬▬▬▬▬▬
▬▬▬▬▬▬▬▬▬▬▬▬▬▬▬▬▬▬▬▬▬▬▬▬▬▬▬▬▬▬▬▬▬▬▬▬▬▬▬▬▬▬▬▬▬▬▬▬▬▬▬▬▬
▬▬▬▬▬▬▬▬▬▬▬▬▬▬▬▬▬▬▬▬▬▬▬▬▬▬▬▬▬▬▬▬▬▬▬▬▬▬▬▬▬▬▬▬▬▬▬▬▬▬▬▬▬
▬▬▬▬▬▬▬▬▬▬▬▬▬▬▬▬▬▬▬▬▬▬▬▬▬▬▬▬▬▬▬▬▬▬▬▬▬▬▬▬▬▬▬▬▬▬▬▬▬▬▬▬▬
▬▬▬▬▬▬▬▬▬▬▬▬▬▬▬▬▬▬▬▬▬▬▬▬▬▬▬▬▬▬▬▬▬▬▬▬▬▬▬▬▬▬▬▬▬▬▬▬▬▬▬▬▬
▬▬▬▬▬▬▬▬▬▬▬▬▬▬▬▬▬▬▬▬▬▬▬▬▬▬▬▬▬▬▬▬▬▬▬▬▬▬▬▬▬▬▬▬▬▬▬▬▬▬▬▬▬

## Slide 7
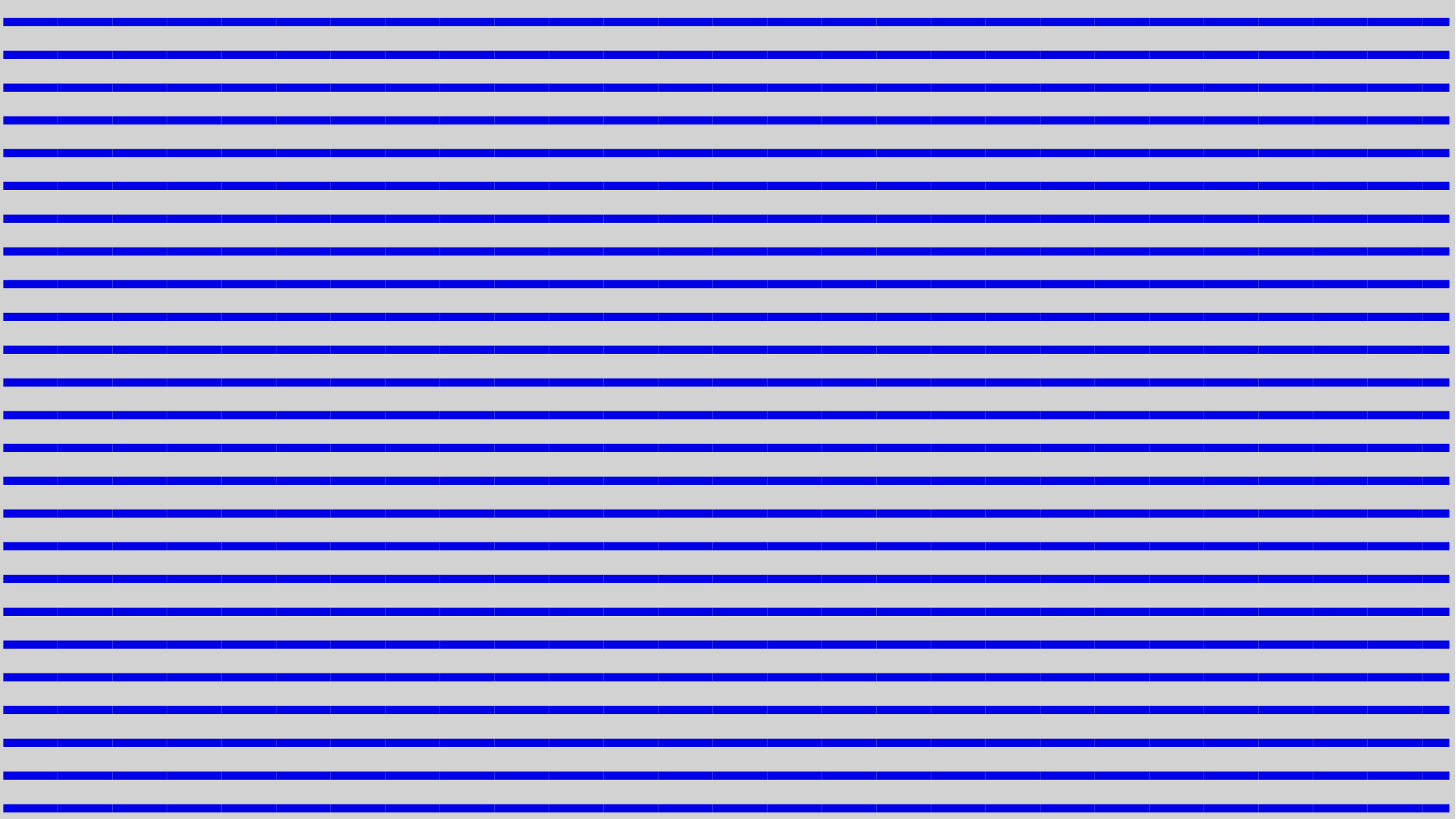

▬▬▬▬▬▬▬▬▬▬▬▬▬▬▬▬▬▬▬▬▬▬▬▬▬▬▬▬▬▬▬▬▬▬▬▬▬▬▬▬▬▬▬▬▬▬▬▬▬▬▬▬▬
▬▬▬▬▬▬▬▬▬▬▬▬▬▬▬▬▬▬▬▬▬▬▬▬▬▬▬▬▬▬▬▬▬▬▬▬▬▬▬▬▬▬▬▬▬▬▬▬▬▬▬▬▬
▬▬▬▬▬▬▬▬▬▬▬▬▬▬▬▬▬▬▬▬▬▬▬▬▬▬▬▬▬▬▬▬▬▬▬▬▬▬▬▬▬▬▬▬▬▬▬▬▬▬▬▬▬
▬▬▬▬▬▬▬▬▬▬▬▬▬▬▬▬▬▬▬▬▬▬▬▬▬▬▬▬▬▬▬▬▬▬▬▬▬▬▬▬▬▬▬▬▬▬▬▬▬▬▬▬▬
▬▬▬▬▬▬▬▬▬▬▬▬▬▬▬▬▬▬▬▬▬▬▬▬▬▬▬▬▬▬▬▬▬▬▬▬▬▬▬▬▬▬▬▬▬▬▬▬▬▬▬▬▬
▬▬▬▬▬▬▬▬▬▬▬▬▬▬▬▬▬▬▬▬▬▬▬▬▬▬▬▬▬▬▬▬▬▬▬▬▬▬▬▬▬▬▬▬▬▬▬▬▬▬▬▬▬
▬▬▬▬▬▬▬▬▬▬▬▬▬▬▬▬▬▬▬▬▬▬▬▬▬▬▬▬▬▬▬▬▬▬▬▬▬▬▬▬▬▬▬▬▬▬▬▬▬▬▬▬▬
▬▬▬▬▬▬▬▬▬▬▬▬▬▬▬▬▬▬▬▬▬▬▬▬▬▬▬▬▬▬▬▬▬▬▬▬▬▬▬▬▬▬▬▬▬▬▬▬▬▬▬▬▬
▬▬▬▬▬▬▬▬▬▬▬▬▬▬▬▬▬▬▬▬▬▬▬▬▬▬▬▬▬▬▬▬▬▬▬▬▬▬▬▬▬▬▬▬▬▬▬▬▬▬▬▬▬
▬▬▬▬▬▬▬▬▬▬▬▬▬▬▬▬▬▬▬▬▬▬▬▬▬▬▬▬▬▬▬▬▬▬▬▬▬▬▬▬▬▬▬▬▬▬▬▬▬▬▬▬▬
▬▬▬▬▬▬▬▬▬▬▬▬▬▬▬▬▬▬▬▬▬▬▬▬▬▬▬▬▬▬▬▬▬▬▬▬▬▬▬▬▬▬▬▬▬▬▬▬▬▬▬▬▬
▬▬▬▬▬▬▬▬▬▬▬▬▬▬▬▬▬▬▬▬▬▬▬▬▬▬▬▬▬▬▬▬▬▬▬▬▬▬▬▬▬▬▬▬▬▬▬▬▬▬▬▬▬
▬▬▬▬▬▬▬▬▬▬▬▬▬▬▬▬▬▬▬▬▬▬▬▬▬▬▬▬▬▬▬▬▬▬▬▬▬▬▬▬▬▬▬▬▬▬▬▬▬▬▬▬▬
▬▬▬▬▬▬▬▬▬▬▬▬▬▬▬▬▬▬▬▬▬▬▬▬▬▬▬▬▬▬▬▬▬▬▬▬▬▬▬▬▬▬▬▬▬▬▬▬▬▬▬▬▬
▬▬▬▬▬▬▬▬▬▬▬▬▬▬▬▬▬▬▬▬▬▬▬▬▬▬▬▬▬▬▬▬▬▬▬▬▬▬▬▬▬▬▬▬▬▬▬▬▬▬▬▬▬
▬▬▬▬▬▬▬▬▬▬▬▬▬▬▬▬▬▬▬▬▬▬▬▬▬▬▬▬▬▬▬▬▬▬▬▬▬▬▬▬▬▬▬▬▬▬▬▬▬▬▬▬▬
▬▬▬▬▬▬▬▬▬▬▬▬▬▬▬▬▬▬▬▬▬▬▬▬▬▬▬▬▬▬▬▬▬▬▬▬▬▬▬▬▬▬▬▬▬▬▬▬▬▬▬▬▬
▬▬▬▬▬▬▬▬▬▬▬▬▬▬▬▬▬▬▬▬▬▬▬▬▬▬▬▬▬▬▬▬▬▬▬▬▬▬▬▬▬▬▬▬▬▬▬▬▬▬▬▬▬
▬▬▬▬▬▬▬▬▬▬▬▬▬▬▬▬▬▬▬▬▬▬▬▬▬▬▬▬▬▬▬▬▬▬▬▬▬▬▬▬▬▬▬▬▬▬▬▬▬▬▬▬▬
▬▬▬▬▬▬▬▬▬▬▬▬▬▬▬▬▬▬▬▬▬▬▬▬▬▬▬▬▬▬▬▬▬▬▬▬▬▬▬▬▬▬▬▬▬▬▬▬▬▬▬▬▬
▬▬▬▬▬▬▬▬▬▬▬▬▬▬▬▬▬▬▬▬▬▬▬▬▬▬▬▬▬▬▬▬▬▬▬▬▬▬▬▬▬▬▬▬▬▬▬▬▬▬▬▬▬
▬▬▬▬▬▬▬▬▬▬▬▬▬▬▬▬▬▬▬▬▬▬▬▬▬▬▬▬▬▬▬▬▬▬▬▬▬▬▬▬▬▬▬▬▬▬▬▬▬▬▬▬▬
▬▬▬▬▬▬▬▬▬▬▬▬▬▬▬▬▬▬▬▬▬▬▬▬▬▬▬▬▬▬▬▬▬▬▬▬▬▬▬▬▬▬▬▬▬▬▬▬▬▬▬▬▬
▬▬▬▬▬▬▬▬▬▬▬▬▬▬▬▬▬▬▬▬▬▬▬▬▬▬▬▬▬▬▬▬▬▬▬▬▬▬▬▬▬▬▬▬▬▬▬▬▬▬▬▬▬
▬▬▬▬▬▬▬▬▬▬▬▬▬▬▬▬▬▬▬▬▬▬▬▬▬▬▬▬▬▬▬▬▬▬▬▬▬▬▬▬▬▬▬▬▬▬▬▬▬▬▬▬▬
▬▬▬▬▬▬▬▬▬▬▬▬▬▬▬▬▬▬▬▬▬▬▬▬▬▬▬▬▬▬▬▬▬▬▬▬▬▬▬▬▬▬▬▬▬▬▬▬▬▬▬▬▬
▬▬▬▬▬▬▬▬▬▬▬▬▬▬▬▬▬▬▬▬▬▬▬▬▬▬▬▬▬▬▬▬▬▬▬▬▬▬▬▬▬▬▬▬▬▬▬▬▬▬▬▬▬
▬▬▬▬▬▬▬▬▬▬▬▬▬▬▬▬▬▬▬▬▬▬▬▬▬▬▬▬▬▬▬▬▬▬▬▬▬▬▬▬▬▬▬▬▬▬▬▬▬▬▬▬▬
▬▬▬▬▬▬▬▬▬▬▬▬▬▬▬▬▬▬▬▬▬▬▬▬▬▬▬▬▬▬▬▬▬▬▬▬▬▬▬▬▬▬▬▬▬▬▬▬▬▬▬▬▬

## Slide 8
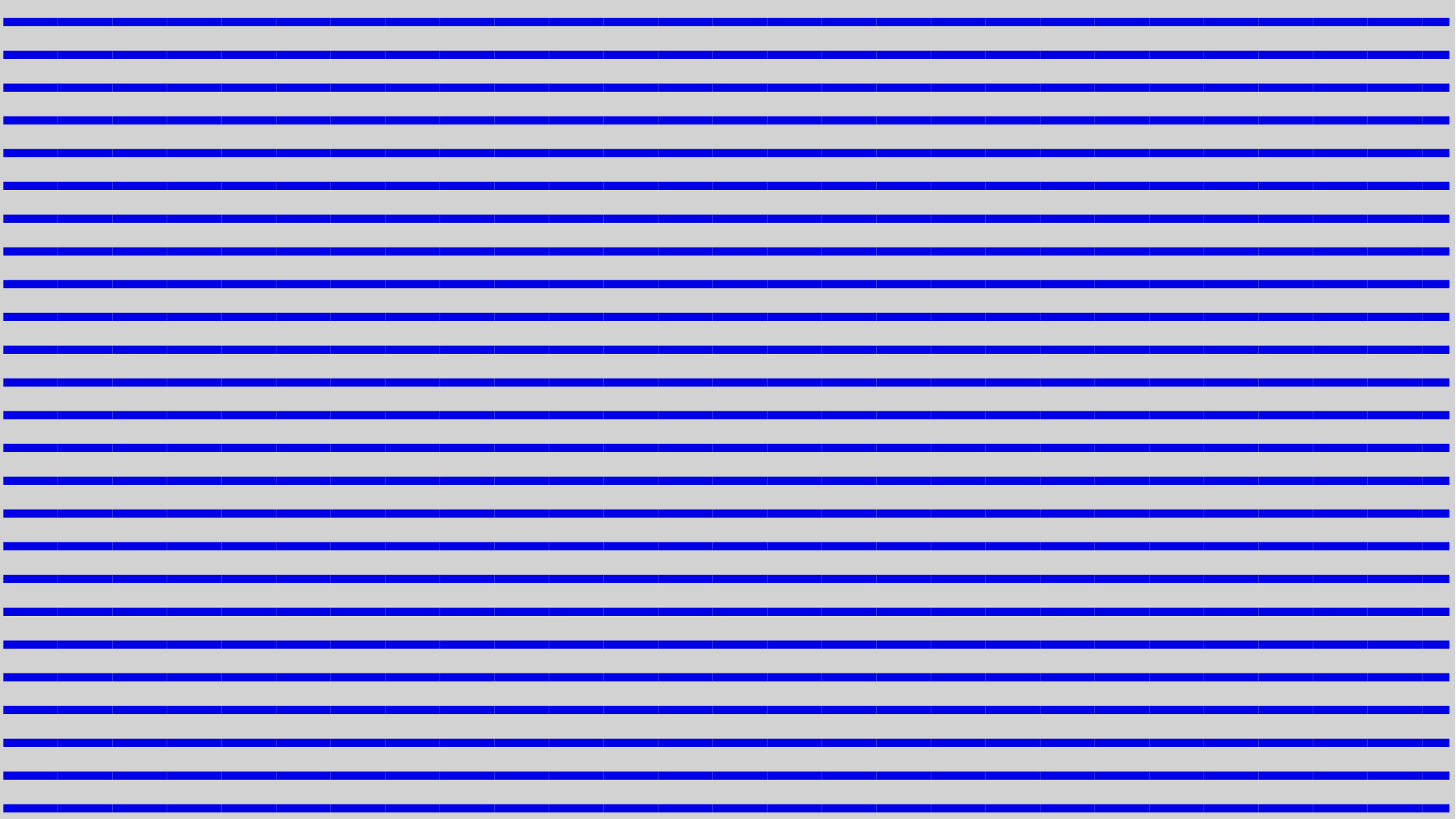

▬▬▬▬▬▬▬▬▬▬▬▬▬▬▬▬▬▬▬▬▬▬▬▬▬▬▬▬▬▬▬▬▬▬▬▬▬▬▬▬▬▬▬▬▬▬▬▬▬▬▬▬▬
▬▬▬▬▬▬▬▬▬▬▬▬▬▬▬▬▬▬▬▬▬▬▬▬▬▬▬▬▬▬▬▬▬▬▬▬▬▬▬▬▬▬▬▬▬▬▬▬▬▬▬▬▬
▬▬▬▬▬▬▬▬▬▬▬▬▬▬▬▬▬▬▬▬▬▬▬▬▬▬▬▬▬▬▬▬▬▬▬▬▬▬▬▬▬▬▬▬▬▬▬▬▬▬▬▬▬
▬▬▬▬▬▬▬▬▬▬▬▬▬▬▬▬▬▬▬▬▬▬▬▬▬▬▬▬▬▬▬▬▬▬▬▬▬▬▬▬▬▬▬▬▬▬▬▬▬▬▬▬▬
▬▬▬▬▬▬▬▬▬▬▬▬▬▬▬▬▬▬▬▬▬▬▬▬▬▬▬▬▬▬▬▬▬▬▬▬▬▬▬▬▬▬▬▬▬▬▬▬▬▬▬▬▬
▬▬▬▬▬▬▬▬▬▬▬▬▬▬▬▬▬▬▬▬▬▬▬▬▬▬▬▬▬▬▬▬▬▬▬▬▬▬▬▬▬▬▬▬▬▬▬▬▬▬▬▬▬
▬▬▬▬▬▬▬▬▬▬▬▬▬▬▬▬▬▬▬▬▬▬▬▬▬▬▬▬▬▬▬▬▬▬▬▬▬▬▬▬▬▬▬▬▬▬▬▬▬▬▬▬▬
▬▬▬▬▬▬▬▬▬▬▬▬▬▬▬▬▬▬▬▬▬▬▬▬▬▬▬▬▬▬▬▬▬▬▬▬▬▬▬▬▬▬▬▬▬▬▬▬▬▬▬▬▬
▬▬▬▬▬▬▬▬▬▬▬▬▬▬▬▬▬▬▬▬▬▬▬▬▬▬▬▬▬▬▬▬▬▬▬▬▬▬▬▬▬▬▬▬▬▬▬▬▬▬▬▬▬
▬▬▬▬▬▬▬▬▬▬▬▬▬▬▬▬▬▬▬▬▬▬▬▬▬▬▬▬▬▬▬▬▬▬▬▬▬▬▬▬▬▬▬▬▬▬▬▬▬▬▬▬▬
▬▬▬▬▬▬▬▬▬▬▬▬▬▬▬▬▬▬▬▬▬▬▬▬▬▬▬▬▬▬▬▬▬▬▬▬▬▬▬▬▬▬▬▬▬▬▬▬▬▬▬▬▬
▬▬▬▬▬▬▬▬▬▬▬▬▬▬▬▬▬▬▬▬▬▬▬▬▬▬▬▬▬▬▬▬▬▬▬▬▬▬▬▬▬▬▬▬▬▬▬▬▬▬▬▬▬
▬▬▬▬▬▬▬▬▬▬▬▬▬▬▬▬▬▬▬▬▬▬▬▬▬▬▬▬▬▬▬▬▬▬▬▬▬▬▬▬▬▬▬▬▬▬▬▬▬▬▬▬▬
▬▬▬▬▬▬▬▬▬▬▬▬▬▬▬▬▬▬▬▬▬▬▬▬▬▬▬▬▬▬▬▬▬▬▬▬▬▬▬▬▬▬▬▬▬▬▬▬▬▬▬▬▬
▬▬▬▬▬▬▬▬▬▬▬▬▬▬▬▬▬▬▬▬▬▬▬▬▬▬▬▬▬▬▬▬▬▬▬▬▬▬▬▬▬▬▬▬▬▬▬▬▬▬▬▬▬
▬▬▬▬▬▬▬▬▬▬▬▬▬▬▬▬▬▬▬▬▬▬▬▬▬▬▬▬▬▬▬▬▬▬▬▬▬▬▬▬▬▬▬▬▬▬▬▬▬▬▬▬▬
▬▬▬▬▬▬▬▬▬▬▬▬▬▬▬▬▬▬▬▬▬▬▬▬▬▬▬▬▬▬▬▬▬▬▬▬▬▬▬▬▬▬▬▬▬▬▬▬▬▬▬▬▬
▬▬▬▬▬▬▬▬▬▬▬▬▬▬▬▬▬▬▬▬▬▬▬▬▬▬▬▬▬▬▬▬▬▬▬▬▬▬▬▬▬▬▬▬▬▬▬▬▬▬▬▬▬
▬▬▬▬▬▬▬▬▬▬▬▬▬▬▬▬▬▬▬▬▬▬▬▬▬▬▬▬▬▬▬▬▬▬▬▬▬▬▬▬▬▬▬▬▬▬▬▬▬▬▬▬▬
▬▬▬▬▬▬▬▬▬▬▬▬▬▬▬▬▬▬▬▬▬▬▬▬▬▬▬▬▬▬▬▬▬▬▬▬▬▬▬▬▬▬▬▬▬▬▬▬▬▬▬▬▬
▬▬▬▬▬▬▬▬▬▬▬▬▬▬▬▬▬▬▬▬▬▬▬▬▬▬▬▬▬▬▬▬▬▬▬▬▬▬▬▬▬▬▬▬▬▬▬▬▬▬▬▬▬
▬▬▬▬▬▬▬▬▬▬▬▬▬▬▬▬▬▬▬▬▬▬▬▬▬▬▬▬▬▬▬▬▬▬▬▬▬▬▬▬▬▬▬▬▬▬▬▬▬▬▬▬▬
▬▬▬▬▬▬▬▬▬▬▬▬▬▬▬▬▬▬▬▬▬▬▬▬▬▬▬▬▬▬▬▬▬▬▬▬▬▬▬▬▬▬▬▬▬▬▬▬▬▬▬▬▬
▬▬▬▬▬▬▬▬▬▬▬▬▬▬▬▬▬▬▬▬▬▬▬▬▬▬▬▬▬▬▬▬▬▬▬▬▬▬▬▬▬▬▬▬▬▬▬▬▬▬▬▬▬
▬▬▬▬▬▬▬▬▬▬▬▬▬▬▬▬▬▬▬▬▬▬▬▬▬▬▬▬▬▬▬▬▬▬▬▬▬▬▬▬▬▬▬▬▬▬▬▬▬▬▬▬▬
▬▬▬▬▬▬▬▬▬▬▬▬▬▬▬▬▬▬▬▬▬▬▬▬▬▬▬▬▬▬▬▬▬▬▬▬▬▬▬▬▬▬▬▬▬▬▬▬▬▬▬▬▬
▬▬▬▬▬▬▬▬▬▬▬▬▬▬▬▬▬▬▬▬▬▬▬▬▬▬▬▬▬▬▬▬▬▬▬▬▬▬▬▬▬▬▬▬▬▬▬▬▬▬▬▬▬
▬▬▬▬▬▬▬▬▬▬▬▬▬▬▬▬▬▬▬▬▬▬▬▬▬▬▬▬▬▬▬▬▬▬▬▬▬▬▬▬▬▬▬▬▬▬▬▬▬▬▬▬▬
▬▬▬▬▬▬▬▬▬▬▬▬▬▬▬▬▬▬▬▬▬▬▬▬▬▬▬▬▬▬▬▬▬▬▬▬▬▬▬▬▬▬▬▬▬▬▬▬▬▬▬▬▬

## Slide 9
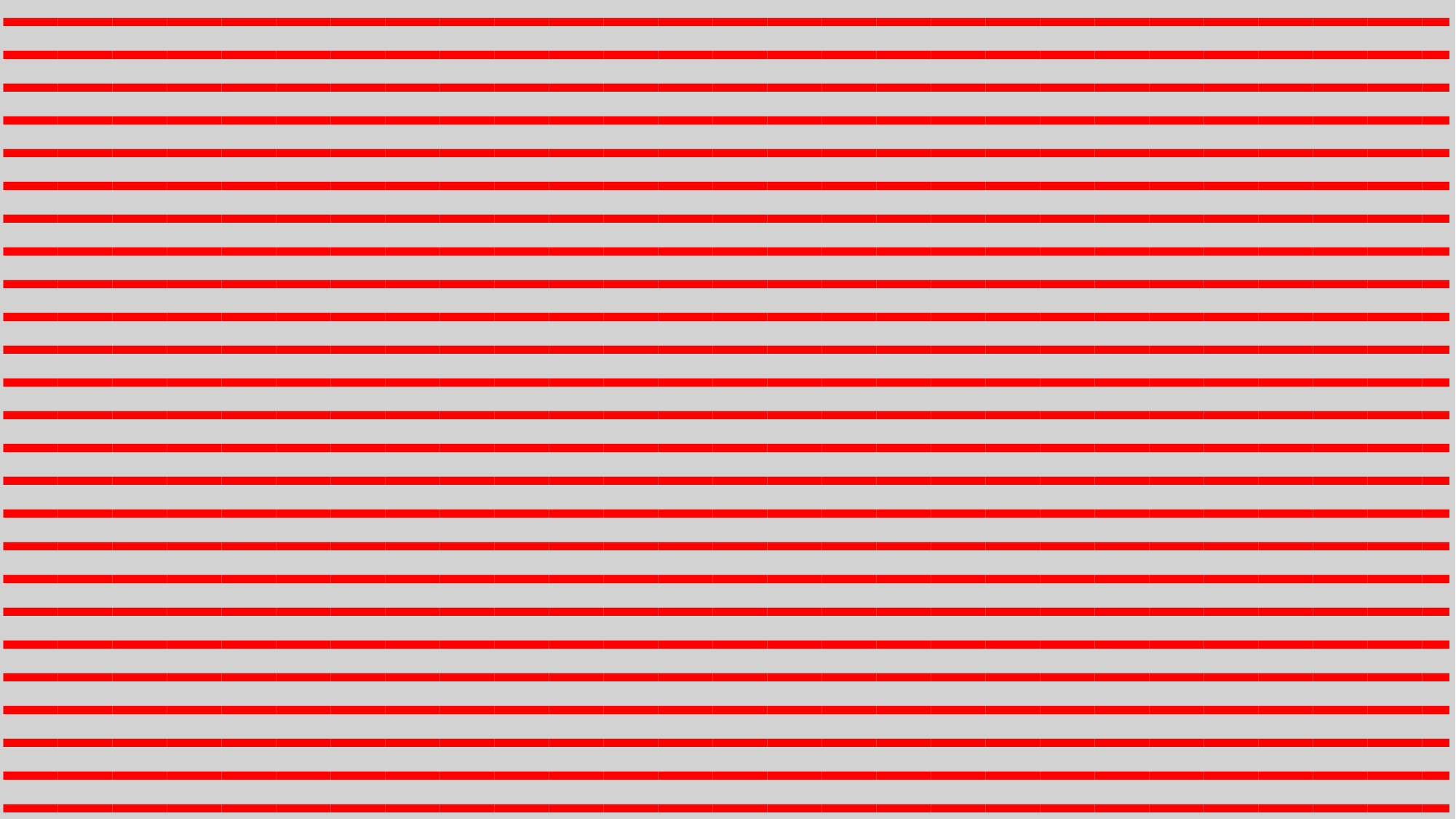

▬▬▬▬▬▬▬▬▬▬▬▬▬▬▬▬▬▬▬▬▬▬▬▬▬▬▬▬▬▬▬▬▬▬▬▬▬▬▬▬▬▬▬▬▬▬▬▬▬▬▬▬▬
▬▬▬▬▬▬▬▬▬▬▬▬▬▬▬▬▬▬▬▬▬▬▬▬▬▬▬▬▬▬▬▬▬▬▬▬▬▬▬▬▬▬▬▬▬▬▬▬▬▬▬▬▬
▬▬▬▬▬▬▬▬▬▬▬▬▬▬▬▬▬▬▬▬▬▬▬▬▬▬▬▬▬▬▬▬▬▬▬▬▬▬▬▬▬▬▬▬▬▬▬▬▬▬▬▬▬
▬▬▬▬▬▬▬▬▬▬▬▬▬▬▬▬▬▬▬▬▬▬▬▬▬▬▬▬▬▬▬▬▬▬▬▬▬▬▬▬▬▬▬▬▬▬▬▬▬▬▬▬▬
▬▬▬▬▬▬▬▬▬▬▬▬▬▬▬▬▬▬▬▬▬▬▬▬▬▬▬▬▬▬▬▬▬▬▬▬▬▬▬▬▬▬▬▬▬▬▬▬▬▬▬▬▬
▬▬▬▬▬▬▬▬▬▬▬▬▬▬▬▬▬▬▬▬▬▬▬▬▬▬▬▬▬▬▬▬▬▬▬▬▬▬▬▬▬▬▬▬▬▬▬▬▬▬▬▬▬
▬▬▬▬▬▬▬▬▬▬▬▬▬▬▬▬▬▬▬▬▬▬▬▬▬▬▬▬▬▬▬▬▬▬▬▬▬▬▬▬▬▬▬▬▬▬▬▬▬▬▬▬▬
▬▬▬▬▬▬▬▬▬▬▬▬▬▬▬▬▬▬▬▬▬▬▬▬▬▬▬▬▬▬▬▬▬▬▬▬▬▬▬▬▬▬▬▬▬▬▬▬▬▬▬▬▬
▬▬▬▬▬▬▬▬▬▬▬▬▬▬▬▬▬▬▬▬▬▬▬▬▬▬▬▬▬▬▬▬▬▬▬▬▬▬▬▬▬▬▬▬▬▬▬▬▬▬▬▬▬
▬▬▬▬▬▬▬▬▬▬▬▬▬▬▬▬▬▬▬▬▬▬▬▬▬▬▬▬▬▬▬▬▬▬▬▬▬▬▬▬▬▬▬▬▬▬▬▬▬▬▬▬▬
▬▬▬▬▬▬▬▬▬▬▬▬▬▬▬▬▬▬▬▬▬▬▬▬▬▬▬▬▬▬▬▬▬▬▬▬▬▬▬▬▬▬▬▬▬▬▬▬▬▬▬▬▬
▬▬▬▬▬▬▬▬▬▬▬▬▬▬▬▬▬▬▬▬▬▬▬▬▬▬▬▬▬▬▬▬▬▬▬▬▬▬▬▬▬▬▬▬▬▬▬▬▬▬▬▬▬
▬▬▬▬▬▬▬▬▬▬▬▬▬▬▬▬▬▬▬▬▬▬▬▬▬▬▬▬▬▬▬▬▬▬▬▬▬▬▬▬▬▬▬▬▬▬▬▬▬▬▬▬▬
▬▬▬▬▬▬▬▬▬▬▬▬▬▬▬▬▬▬▬▬▬▬▬▬▬▬▬▬▬▬▬▬▬▬▬▬▬▬▬▬▬▬▬▬▬▬▬▬▬▬▬▬▬
▬▬▬▬▬▬▬▬▬▬▬▬▬▬▬▬▬▬▬▬▬▬▬▬▬▬▬▬▬▬▬▬▬▬▬▬▬▬▬▬▬▬▬▬▬▬▬▬▬▬▬▬▬
▬▬▬▬▬▬▬▬▬▬▬▬▬▬▬▬▬▬▬▬▬▬▬▬▬▬▬▬▬▬▬▬▬▬▬▬▬▬▬▬▬▬▬▬▬▬▬▬▬▬▬▬▬
▬▬▬▬▬▬▬▬▬▬▬▬▬▬▬▬▬▬▬▬▬▬▬▬▬▬▬▬▬▬▬▬▬▬▬▬▬▬▬▬▬▬▬▬▬▬▬▬▬▬▬▬▬
▬▬▬▬▬▬▬▬▬▬▬▬▬▬▬▬▬▬▬▬▬▬▬▬▬▬▬▬▬▬▬▬▬▬▬▬▬▬▬▬▬▬▬▬▬▬▬▬▬▬▬▬▬
▬▬▬▬▬▬▬▬▬▬▬▬▬▬▬▬▬▬▬▬▬▬▬▬▬▬▬▬▬▬▬▬▬▬▬▬▬▬▬▬▬▬▬▬▬▬▬▬▬▬▬▬▬
▬▬▬▬▬▬▬▬▬▬▬▬▬▬▬▬▬▬▬▬▬▬▬▬▬▬▬▬▬▬▬▬▬▬▬▬▬▬▬▬▬▬▬▬▬▬▬▬▬▬▬▬▬
▬▬▬▬▬▬▬▬▬▬▬▬▬▬▬▬▬▬▬▬▬▬▬▬▬▬▬▬▬▬▬▬▬▬▬▬▬▬▬▬▬▬▬▬▬▬▬▬▬▬▬▬▬
▬▬▬▬▬▬▬▬▬▬▬▬▬▬▬▬▬▬▬▬▬▬▬▬▬▬▬▬▬▬▬▬▬▬▬▬▬▬▬▬▬▬▬▬▬▬▬▬▬▬▬▬▬
▬▬▬▬▬▬▬▬▬▬▬▬▬▬▬▬▬▬▬▬▬▬▬▬▬▬▬▬▬▬▬▬▬▬▬▬▬▬▬▬▬▬▬▬▬▬▬▬▬▬▬▬▬
▬▬▬▬▬▬▬▬▬▬▬▬▬▬▬▬▬▬▬▬▬▬▬▬▬▬▬▬▬▬▬▬▬▬▬▬▬▬▬▬▬▬▬▬▬▬▬▬▬▬▬▬▬
▬▬▬▬▬▬▬▬▬▬▬▬▬▬▬▬▬▬▬▬▬▬▬▬▬▬▬▬▬▬▬▬▬▬▬▬▬▬▬▬▬▬▬▬▬▬▬▬▬▬▬▬▬
▬▬▬▬▬▬▬▬▬▬▬▬▬▬▬▬▬▬▬▬▬▬▬▬▬▬▬▬▬▬▬▬▬▬▬▬▬▬▬▬▬▬▬▬▬▬▬▬▬▬▬▬▬
▬▬▬▬▬▬▬▬▬▬▬▬▬▬▬▬▬▬▬▬▬▬▬▬▬▬▬▬▬▬▬▬▬▬▬▬▬▬▬▬▬▬▬▬▬▬▬▬▬▬▬▬▬
▬▬▬▬▬▬▬▬▬▬▬▬▬▬▬▬▬▬▬▬▬▬▬▬▬▬▬▬▬▬▬▬▬▬▬▬▬▬▬▬▬▬▬▬▬▬▬▬▬▬▬▬▬
▬▬▬▬▬▬▬▬▬▬▬▬▬▬▬▬▬▬▬▬▬▬▬▬▬▬▬▬▬▬▬▬▬▬▬▬▬▬▬▬▬▬▬▬▬▬▬▬▬▬▬▬▬

## Slide 10
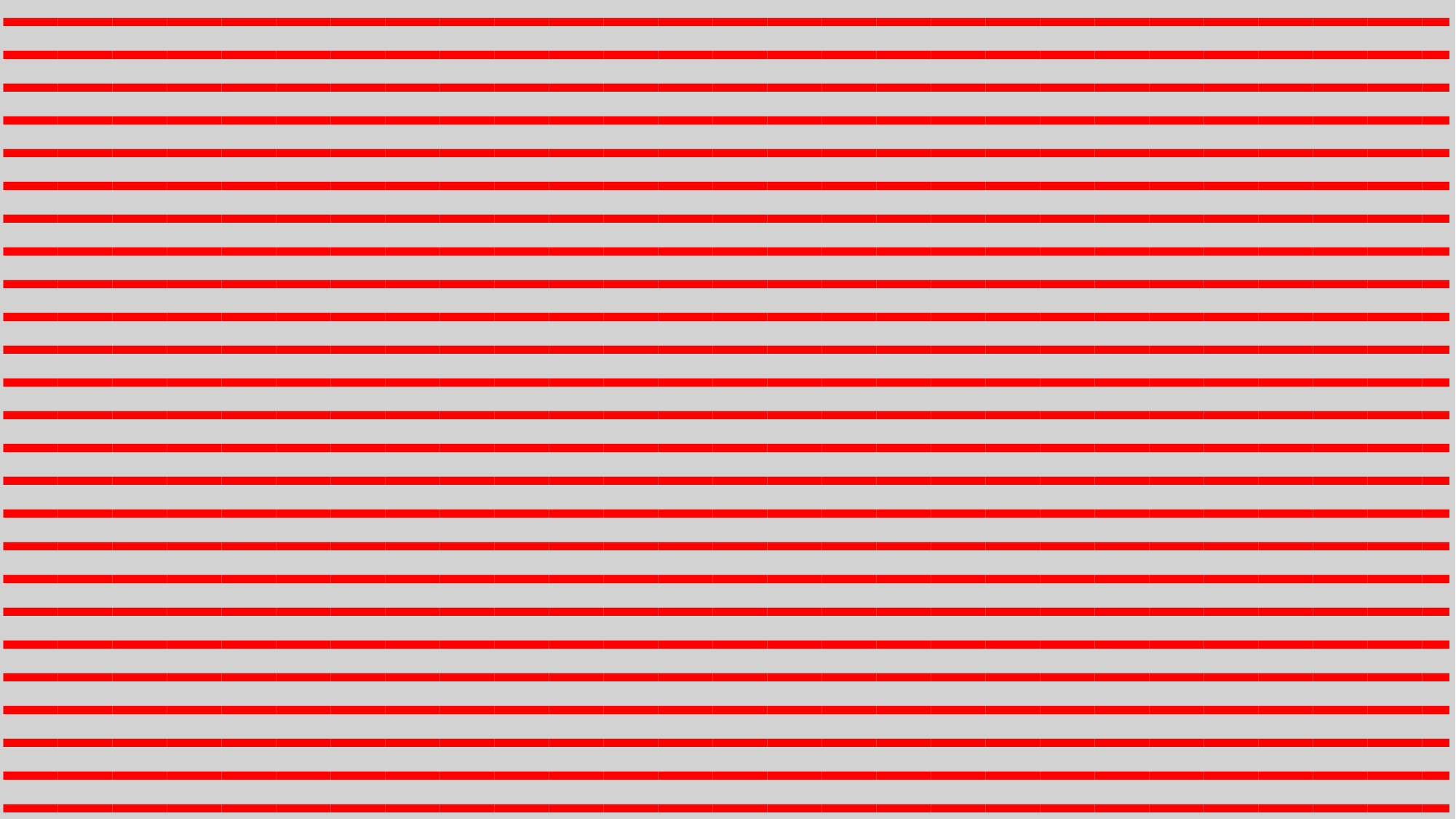

▬▬▬▬▬▬▬▬▬▬▬▬▬▬▬▬▬▬▬▬▬▬▬▬▬▬▬▬▬▬▬▬▬▬▬▬▬▬▬▬▬▬▬▬▬▬▬▬▬▬▬▬▬
▬▬▬▬▬▬▬▬▬▬▬▬▬▬▬▬▬▬▬▬▬▬▬▬▬▬▬▬▬▬▬▬▬▬▬▬▬▬▬▬▬▬▬▬▬▬▬▬▬▬▬▬▬
▬▬▬▬▬▬▬▬▬▬▬▬▬▬▬▬▬▬▬▬▬▬▬▬▬▬▬▬▬▬▬▬▬▬▬▬▬▬▬▬▬▬▬▬▬▬▬▬▬▬▬▬▬
▬▬▬▬▬▬▬▬▬▬▬▬▬▬▬▬▬▬▬▬▬▬▬▬▬▬▬▬▬▬▬▬▬▬▬▬▬▬▬▬▬▬▬▬▬▬▬▬▬▬▬▬▬
▬▬▬▬▬▬▬▬▬▬▬▬▬▬▬▬▬▬▬▬▬▬▬▬▬▬▬▬▬▬▬▬▬▬▬▬▬▬▬▬▬▬▬▬▬▬▬▬▬▬▬▬▬
▬▬▬▬▬▬▬▬▬▬▬▬▬▬▬▬▬▬▬▬▬▬▬▬▬▬▬▬▬▬▬▬▬▬▬▬▬▬▬▬▬▬▬▬▬▬▬▬▬▬▬▬▬
▬▬▬▬▬▬▬▬▬▬▬▬▬▬▬▬▬▬▬▬▬▬▬▬▬▬▬▬▬▬▬▬▬▬▬▬▬▬▬▬▬▬▬▬▬▬▬▬▬▬▬▬▬
▬▬▬▬▬▬▬▬▬▬▬▬▬▬▬▬▬▬▬▬▬▬▬▬▬▬▬▬▬▬▬▬▬▬▬▬▬▬▬▬▬▬▬▬▬▬▬▬▬▬▬▬▬
▬▬▬▬▬▬▬▬▬▬▬▬▬▬▬▬▬▬▬▬▬▬▬▬▬▬▬▬▬▬▬▬▬▬▬▬▬▬▬▬▬▬▬▬▬▬▬▬▬▬▬▬▬
▬▬▬▬▬▬▬▬▬▬▬▬▬▬▬▬▬▬▬▬▬▬▬▬▬▬▬▬▬▬▬▬▬▬▬▬▬▬▬▬▬▬▬▬▬▬▬▬▬▬▬▬▬
▬▬▬▬▬▬▬▬▬▬▬▬▬▬▬▬▬▬▬▬▬▬▬▬▬▬▬▬▬▬▬▬▬▬▬▬▬▬▬▬▬▬▬▬▬▬▬▬▬▬▬▬▬
▬▬▬▬▬▬▬▬▬▬▬▬▬▬▬▬▬▬▬▬▬▬▬▬▬▬▬▬▬▬▬▬▬▬▬▬▬▬▬▬▬▬▬▬▬▬▬▬▬▬▬▬▬
▬▬▬▬▬▬▬▬▬▬▬▬▬▬▬▬▬▬▬▬▬▬▬▬▬▬▬▬▬▬▬▬▬▬▬▬▬▬▬▬▬▬▬▬▬▬▬▬▬▬▬▬▬
▬▬▬▬▬▬▬▬▬▬▬▬▬▬▬▬▬▬▬▬▬▬▬▬▬▬▬▬▬▬▬▬▬▬▬▬▬▬▬▬▬▬▬▬▬▬▬▬▬▬▬▬▬
▬▬▬▬▬▬▬▬▬▬▬▬▬▬▬▬▬▬▬▬▬▬▬▬▬▬▬▬▬▬▬▬▬▬▬▬▬▬▬▬▬▬▬▬▬▬▬▬▬▬▬▬▬
▬▬▬▬▬▬▬▬▬▬▬▬▬▬▬▬▬▬▬▬▬▬▬▬▬▬▬▬▬▬▬▬▬▬▬▬▬▬▬▬▬▬▬▬▬▬▬▬▬▬▬▬▬
▬▬▬▬▬▬▬▬▬▬▬▬▬▬▬▬▬▬▬▬▬▬▬▬▬▬▬▬▬▬▬▬▬▬▬▬▬▬▬▬▬▬▬▬▬▬▬▬▬▬▬▬▬
▬▬▬▬▬▬▬▬▬▬▬▬▬▬▬▬▬▬▬▬▬▬▬▬▬▬▬▬▬▬▬▬▬▬▬▬▬▬▬▬▬▬▬▬▬▬▬▬▬▬▬▬▬
▬▬▬▬▬▬▬▬▬▬▬▬▬▬▬▬▬▬▬▬▬▬▬▬▬▬▬▬▬▬▬▬▬▬▬▬▬▬▬▬▬▬▬▬▬▬▬▬▬▬▬▬▬
▬▬▬▬▬▬▬▬▬▬▬▬▬▬▬▬▬▬▬▬▬▬▬▬▬▬▬▬▬▬▬▬▬▬▬▬▬▬▬▬▬▬▬▬▬▬▬▬▬▬▬▬▬
▬▬▬▬▬▬▬▬▬▬▬▬▬▬▬▬▬▬▬▬▬▬▬▬▬▬▬▬▬▬▬▬▬▬▬▬▬▬▬▬▬▬▬▬▬▬▬▬▬▬▬▬▬
▬▬▬▬▬▬▬▬▬▬▬▬▬▬▬▬▬▬▬▬▬▬▬▬▬▬▬▬▬▬▬▬▬▬▬▬▬▬▬▬▬▬▬▬▬▬▬▬▬▬▬▬▬
▬▬▬▬▬▬▬▬▬▬▬▬▬▬▬▬▬▬▬▬▬▬▬▬▬▬▬▬▬▬▬▬▬▬▬▬▬▬▬▬▬▬▬▬▬▬▬▬▬▬▬▬▬
▬▬▬▬▬▬▬▬▬▬▬▬▬▬▬▬▬▬▬▬▬▬▬▬▬▬▬▬▬▬▬▬▬▬▬▬▬▬▬▬▬▬▬▬▬▬▬▬▬▬▬▬▬
▬▬▬▬▬▬▬▬▬▬▬▬▬▬▬▬▬▬▬▬▬▬▬▬▬▬▬▬▬▬▬▬▬▬▬▬▬▬▬▬▬▬▬▬▬▬▬▬▬▬▬▬▬
▬▬▬▬▬▬▬▬▬▬▬▬▬▬▬▬▬▬▬▬▬▬▬▬▬▬▬▬▬▬▬▬▬▬▬▬▬▬▬▬▬▬▬▬▬▬▬▬▬▬▬▬▬
▬▬▬▬▬▬▬▬▬▬▬▬▬▬▬▬▬▬▬▬▬▬▬▬▬▬▬▬▬▬▬▬▬▬▬▬▬▬▬▬▬▬▬▬▬▬▬▬▬▬▬▬▬
▬▬▬▬▬▬▬▬▬▬▬▬▬▬▬▬▬▬▬▬▬▬▬▬▬▬▬▬▬▬▬▬▬▬▬▬▬▬▬▬▬▬▬▬▬▬▬▬▬▬▬▬▬
▬▬▬▬▬▬▬▬▬▬▬▬▬▬▬▬▬▬▬▬▬▬▬▬▬▬▬▬▬▬▬▬▬▬▬▬▬▬▬▬▬▬▬▬▬▬▬▬▬▬▬▬▬

## Slide 11
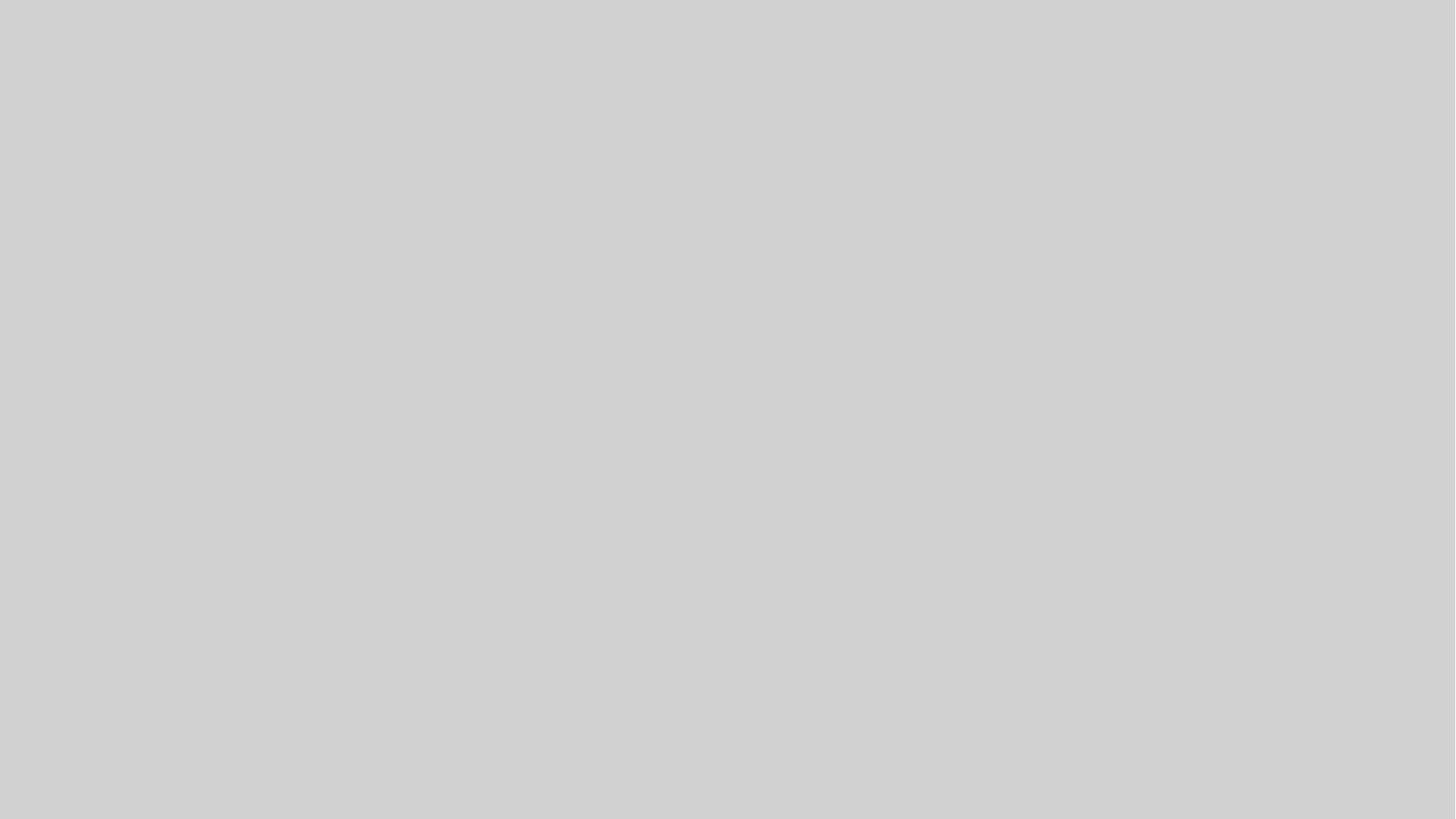

## Slide 12
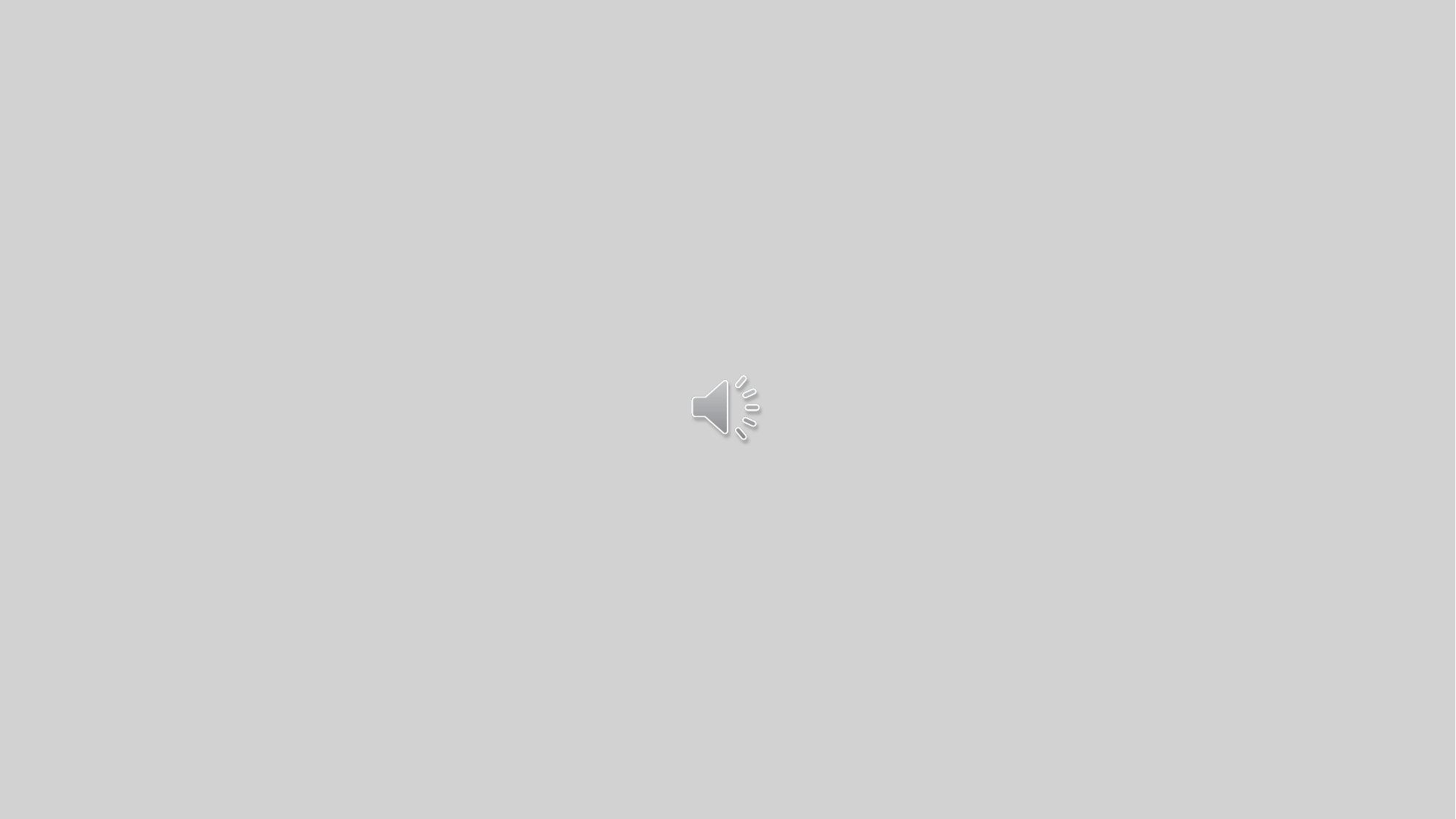

## Slide 13
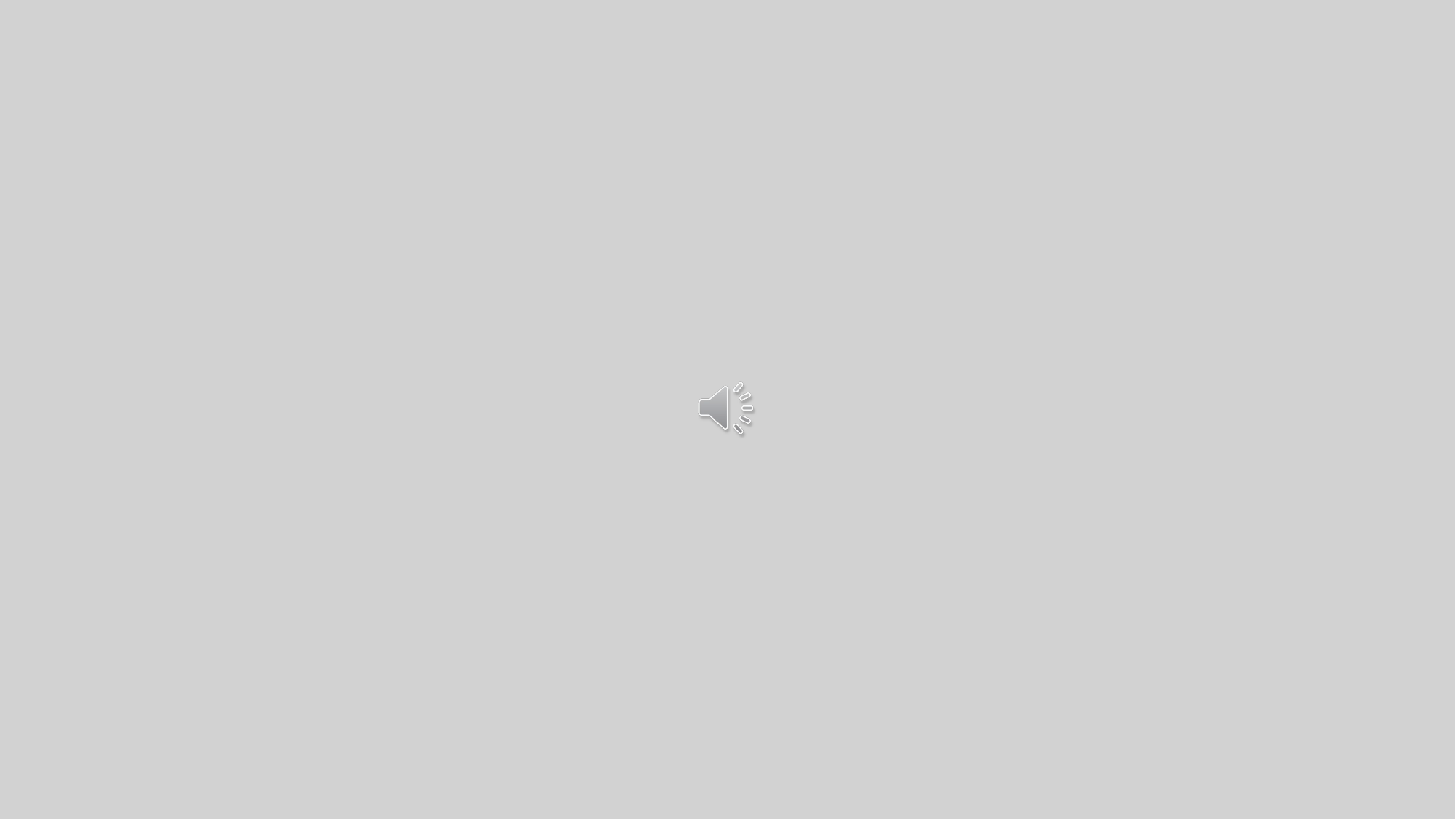

## Slide 14
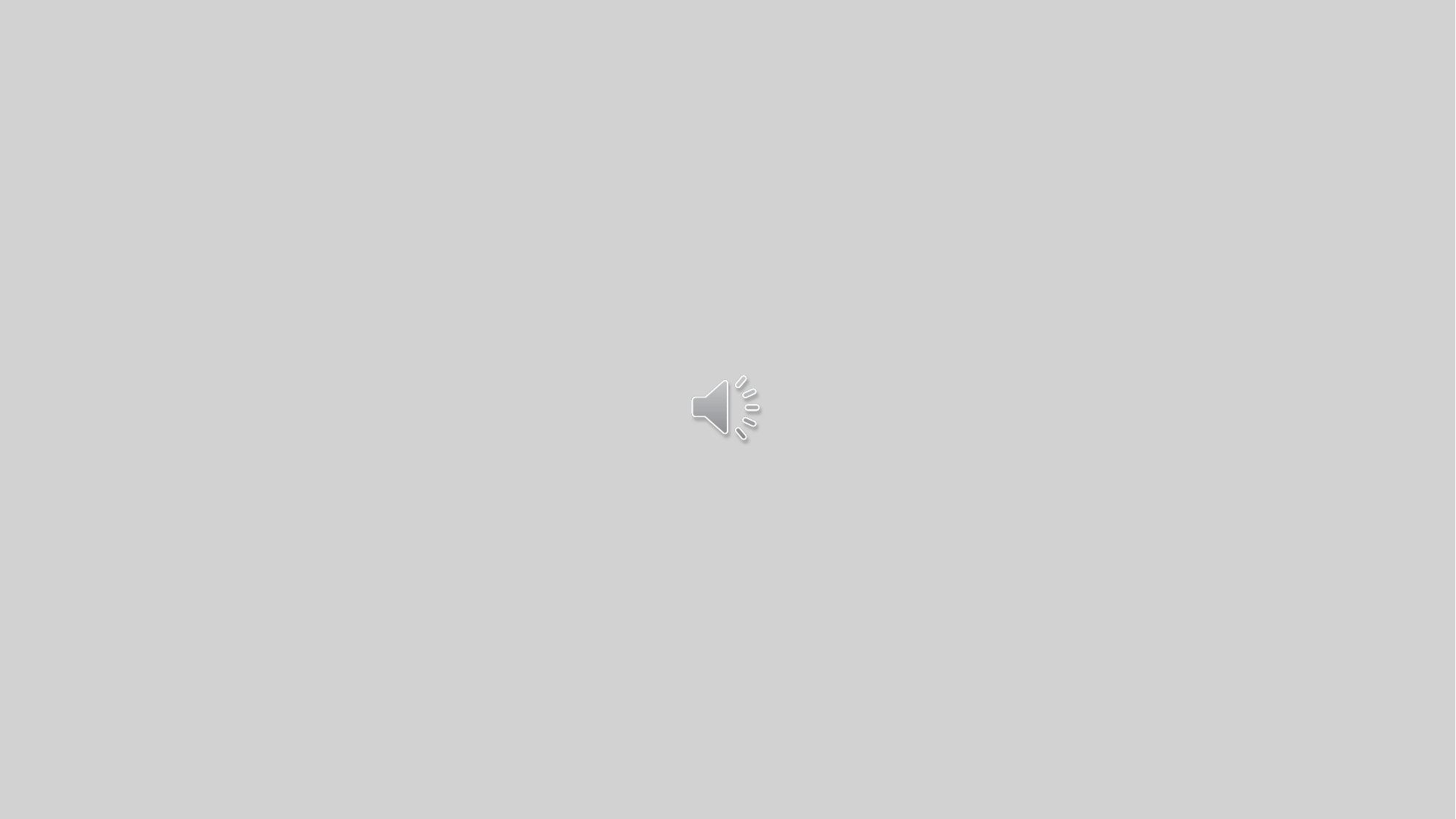

Supplement: Supplementary file 1 — Supplementary Information 1. [file 41598_2022_10133_MOESM1_ESM.ppt]
