# Supplementary figures and images for: Novel use of FDA-approved drugs identified by cluster analysis of behavioral profiles
Source: Sci Rep. 2022 Apr 21;12:6120. doi: 10.1038/s41598-022-10133-y (PMC9023506; doi:10.1038/s41598-022-10133-y)

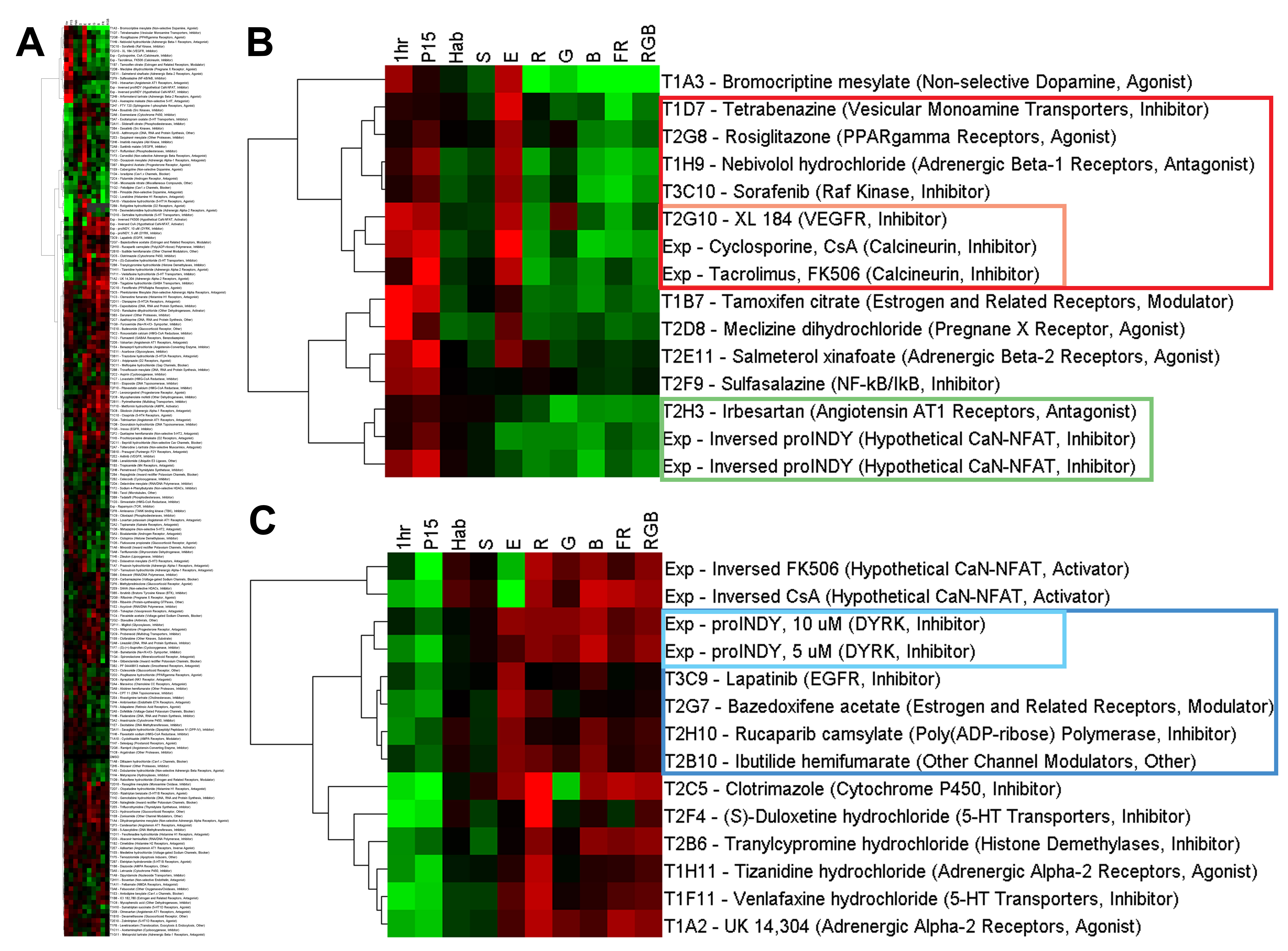

Supplement: Supplementary file 6 — Supplementary Information 6. [file 41598_2022_10133_MOESM6_ESM.jpg]
